# eIF3 engages with 3’-UTR termini of highly translated mRNAs in neural progenitor cells

**DOI:** 10.1101/2023.11.11.566681

**Published:** 2023-11-11

**Authors:** Santi Mestre-Fos, Lucas Ferguson, Marena Trinidad, Nicholas T. Ingolia, Jamie H. D. Cate

**Affiliations:** 1 Innovative Genomics Institute, University of California, Berkeley, CA, USA; 2 Department of Molecular and Cell Biology, University of California, Berkeley, CA, USA; 3 Center for Computational Biology, University of California, Berkeley, CA, USA; 4 Howard Hughes Medical Institute, University of California, Berkeley, CA, USA; 5 California Institute for Quantitative Biosciences, University of California, Berkeley, CA, USA; 6 Department of Chemistry, University of California, Berkeley, CA, USA

## Abstract

Stem cell differentiation involves a global increase in protein synthesis to meet the demands of specialized cell types. However, the molecular mechanisms underlying this translational burst and the involvement of initiation factors remains largely unknown. Here, we investigate the roles of eukaryotic initiation factor 3 (eIF3) in early differentiation of human pluripotent stem cell (hPSC)-derived neural progenitor cells (NPCs). Using Quick-irCLIP and alternative polyadenylation (APA) Seq, we show eIF3 crosslinks to many neurologically relevant mRNAs in NPCs. Our data reveal eIF3 predominantly interacts with 3’ untranslated region (3’-UTR) termini of multiple mRNA isoforms, adjacent to the poly(A) tail. High eIF3 crosslinking at 3’-UTR termini of mRNAs correlates with high translational activity, as determined by ribosome profiling. We identify the transcriptional regulator inhibitor of DNA binding 2 (*ID2*) mRNA as a case in which active translation levels and eIF3 crosslinking are dramatically increased upon early NPC differentiation. Furthermore, we find that eIF3 engagement at 3’-UTR ends is dependent on polyadenylation. The results presented here show that eIF3 engages with 3’-UTR termini of highly translated mRNAs, supporting a role of mRNA circularization in the mechanisms governing mRNA translation in NPCs.

## INTRODUCTION

Stem cells are a group of diverse cells that are characterized by their ability to self-renew and differentiate into multiple cell types. In their quiescent state, they present low protein synthesis levels. However, upon differentiation they exhibit a global increase in protein synthesis that results in a drastic change in the proteome composition in order to be able to cope with the needs of the new specialized progenitor cells^1^. This phenomenon was first observed in mouse embryonic stem cells (ESCs)^2^ and was later confirmed in studies performed with quiescent hematopoietic stem cells (HSCs)^3^, quiescent neural stem cells (NSCs)^4^, quiescent hair follicle stem cells (HFSCs)^5^, and quiescent muscle stem cells^6^, suggesting that it is a common characteristic of quiescent stem cells. Since initiation is translation’s rate-limiting step, it must be highly regulated to maintain quiescence and to regulate the global increase in translation that occurs during the initial steps of stem cell differentiation. However, the roles that initiation factors play in quiescent and newly differentiated stem cells are poorly understood.

Eukaryotic initiation factor 3 (eIF3) is the largest of all initiation factors in eukaryotes. In humans, it is composed of 13 subunits that assemble to help ribosomes position at the start codon of mRNAs^7^. However, several non-canonical functions of eIF3 have been recently discovered. Photoactivatable ribonucleoside-enhanced crosslinking and immunoprecipitation (PAR-CLIP)^8^ experiments performed in human embryonic kidney (HEK) 293T cells uncovered regulatory roles of eIF3 in the translation of a specific pool of mRNAs^9^ and also revealed that eIF3 subunit d (EIF3D) activates the translation of *JUN* mRNA by binding to its 5’ cap^10^. Intriguingly, the pool of mRNAs regulated by eIF3 varies dramatically across different types of cells. eIF3 PAR-CLIP experiments performed in Jurkat T cells determined that robust T cell activation requires the direct interaction of eIF3 with the mRNAs encoding for the human T cell receptor subunits α and β, a phenomenon that was also observed in primary T cells^11^. These results indicate that eIF3 serves different regulatory roles depending on specific cellular environments.

Here, we derived neural progenitor cells (NPCs) from human pluripotent stem cells (hPSCs) and committed them to a forebrain neuron fate to look for the differentiation-dependent global increase in protein synthesis. We then identified the mRNAs that crosslink to eIF3 during this translational burst as well as those that crosslink to eIF3 in undifferentiated NPCs. We found that eIF3 predominantly crosslinks to mRNA 3’-untranslated regions (3’-UTRs). By mapping all polyadenylation events in NPCs using next-generation sequencing, we found that eIF3 crosslinking at 3’-UTRs occurs at 3’-UTR termini of multiple mRNA isoforms, adjacent to polyadenylated (polyA) tails. Our results also show that eIF3 engagement at the 3’-UTR terminus of the mRNA encoding for the inhibitor of DNA binding 2 (ID2) transcription regulator is dramatically increased upon NPC differentiation. Using ribosome profiling, we found that this increase in eIF3 accumulation at 3’-UTR termini is due to increased levels of *ID2* mRNA active translation. At a transcriptome-wide level, we also observe that increased eIF3 crosslinking at 3’-UTR termini correlates with an increased level of ribosome footprints (RPFs) on mRNAs. Lastly, we found that eIF3 crosslinking at 3’-UTR ends is only observed in polyadenylated transcripts, but is independent of interactions between eIF3 and poly(A)-binding proteins, as inferred from eIF3 immunoprecipitation experiments. Overall, our results show that high levels of active translation result in eIF3 engagement at 3’-UTR termini. Given that eIF3 is an initiation factor that is known to associate with 5’-UTRs to promote translation initiation, our experimental observation of eIF3 interactions with 3’-UTR ends suggests that eIF3 plays an important role in mRNA circularization by promoting the communication between 5’ and 3’ ends of actively translated mRNAs.

## RESULTS

### Generation of hPSC-derived NPCs

**Figure 1.**
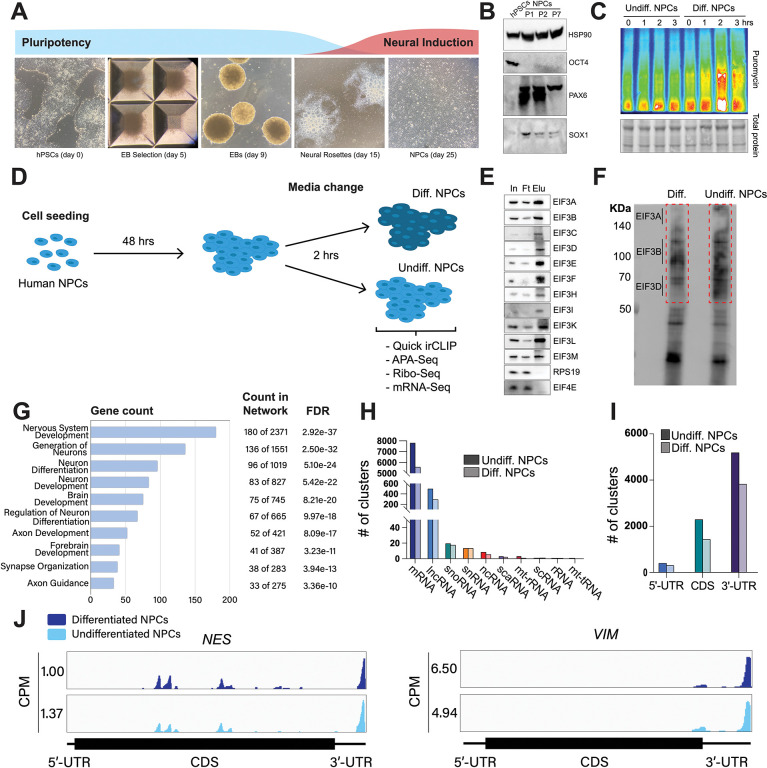
Analysis of Quick irCLIP of eIF3 to RNAs in undifferentiated and differentiated neural progenitor cells. **A)** Generation of NPCs from hPSCs by embryoid body (EB) and neural rosette selections. **B)** Western blots of neural markers Pax6 and Sox1 and pluripotent marker Oct4 from hPSCs and hPSC-derived NPCs (NPCs corresponding to passages 1, 2 and 7 are shown). **C)** Western blots of puromycin-treated (15 min) NPCs. Total protein stain is shown as loading control. Differentiated NPCs were treated with forebrain neuron differentiation medium for the indicated time points. Undifferentiated NPCs were treated with NPC medium for the indicated time points. **D)** Schematic of the NPC treatment performed in the Quick-irCLIP, APA-Seq, Ribosome profiling, and mRNA-Seq experiments. Treatment consisted of change of media and incubation at 37 °C for 2 hrs. Differentiation media was used for differentiated (Diff.) NPCs and Basal media for undifferentiated (Undiff.) NPCs. **E)** Immunoprecipitation samples of assembled eIF3 complexes from NPCs. 5% inputs (In), flowthroughs (Ft), and eluates (Elu) are shown. Western blot for subunit EIF3G is not shown because we could not identify an effective antibody for its detection. EIF3J, not shown, is usually dissociated from the eIF3 complex upon immunoprecipitation. However, we detected EIF3G by mass spectrometry in the IPs (Table S1). **F)** Infrared (IR) image of IR dye-labeled, eIF3 UV-crosslinked RNA transcripts from Diff. and Undiff. NPCs. Regions marked with red boxes, which correspond to subunits EIF3A through EIF3D, were excised from the blot. **G)** Biological Function enrichment determined using the STRING database for the undifferentiated NPC biological replicates of the EIF3A/B/C/D Quick-irCLIP libraries, using the top 500 mRNA hits from the crosslinking analysis. **H)** Categories of RNAs crosslinked to eIF3 in undifferentiated and differentiated NPCs. **I)** mRNA regions that crosslink to eIF3 in undifferentiated and differentiated NPCs. **J)** Crosslinking of eIF3 across the transcripts of *VIM* and *NES* mRNAs in differentiated and undifferentiated NPCs. Read coverage is provided in Counts Per Million (CPM).

We differentiated hPSCs toward NPCs by embryoid body (EB) formation followed by neural rosette selection, giving rise to NPCs after ~3 weeks (Figure 1A). Successful NPC differentiation was assessed by western blotting. NPCs cultured for several passages (up to passage 7) presented an increase in the expression of neural markers Pax6 and Sox1 and the loss of the pluripotent marker Oct4 (Figure 1B).

### Early NPC differentiation results in a global increase in protein synthesis

To find the translational burst that occurs during the initial steps of stem cell differentiation, we differentiated NPCs toward a forebrain neuron fate using a dual-SMAD inhibition protocol^12^ and collected them at different time points that ranged from 1 hour to several days. In order to study active translation levels, prior to cell collection, we treated cells for a short time with puromycin, a tRNA analog that gets incorporated into the C-terminus of elongating nascent chains, releasing them from translating ribosomes. By immunoblotting with puromycin antibodies, puromycin-containing nascent peptides can be detected, reporting on active levels of protein translation. Our western blotting results show that NPCs treated for 2 hours with forebrain neuron differentiation medium present a significant increase in puromycin incorporation into nascent chains compared to NPCs treated for the same amount of time with medium that keeps them in their undifferentiated state (Figure 1C), indicating a differentiation-dependent global increase in protein synthesis.

### eIF3 crosslinks to 5’- and 3’-UTRs of neurologically-relevant mRNAs

To identify the RNA transcripts that interact with eIF3 during the observed translational burst in differentiated NPCs, we performed Quick-irCLIP^13^. We selected the aforementioned 2-hour treatment timepoint and immunoprecipitated eIF3 complexes from early differentiated NPCs that had started the differentiation pathway toward forebrain neurons (differentiated NPCs) and NPCs that we kept in their progenitor state for the same amount of time (undifferentiated NPCs) (Figure 1D). After UV crosslinking the cells, we treated cell lysates with RNAse I, followed by eIF3 immunoprecipitation, dephosphorylation of the protein-bound transcripts, IR adaptor ligation, and RNA-protein complex visualization via SDS-PAGE. We assessed the success of eIF3 immunoprecipitation by western blotting (Figure 1E) and mass spectrometry (Table S1). The previous PAR-CLIP studies performed with the eIF3 complex in HEK293T and Jurkat cells identified eIF3 subunits EIF3A, EIF3B, and EIF3D, and to a lesser extent, EIF3G as the four subunits of the complex presenting significant amounts of RNA crosslinks^9,11^. Consequently, here we excised the RNA smears that appeared in the regions of subunits EIF3A/B/C/D (from ~170 kDa to ~65 KDa) (Figure 1F).

Using the STRING database^14^ for the top 500 transcripts that crosslink to eIF3, we observed highly similar sets of mRNAs in undifferentiated and differentiated NPCs. We observe a significant enrichment in neurologically-relevant biological processes, such as ‘generation of neurons’, ‘neuron differentiation’, ‘neuron projection development’, and ‘axon development’ (Figure 1G). To determine which top eIF3-binding transcripts are common between NPCs and the previous Jurkat T cell^11^ and HEK293T cell^9^ crosslinking studies, we compared the top ~200 eIF3-crosslinked transcripts between the three cell lines. We found that only 12 are common between NPCs and Jurkat T cells and only 8 are common between NPCs and HEK293T, with no common hits between the 3 cell types (Figure S1).

Both in differentiated and undifferentiated NPCs, eIF3 predominantly (>90%) crosslinks to mRNAs over other non-coding RNAs such as lncRNAs and snoRNAs (Figure 1H). Within mRNA regions, eIF3 primarily interacts with 3’-UTRs and, to a lesser extent, with CDS regions and 5’-UTRs (Figure 1I). Our Quick-irCLIP experiment in NPCs identified several eIF3 crosslinks located at 5’-UTR regions that were also observed in the previous eIF3 PAR-CLIP experiment performed in HEK293T^9^, such as *CCND2* and *TUBB* mRNAs (Figure S2). The distinctive ‘pan-mRNA’ pattern observed in the eIF3 PAR-CLIP performed in Jurkat T cells^11^ is not observed in NPCs. Notably, here we observe an enrichment of eIF3 crosslinks at 3’-UTRs of transcripts encoding neurologically-relevant proteins, such as *VIM* and *NES* (Figure 1J), as well as highly abundant mRNAs, such as *ACTG1* and *FTL* (Figure S3). Overall, we observe similar eIF3 crosslinking levels to transcripts in differentiated and undifferentiated NPCs (*VIM* and *NES* are shown as examples in Figure 1J).

### eIF3 3’-UTR crosslinking events map to 3’-UTR termini of multiple isoforms, upstream of poly(A) tails

**Figure 2.**
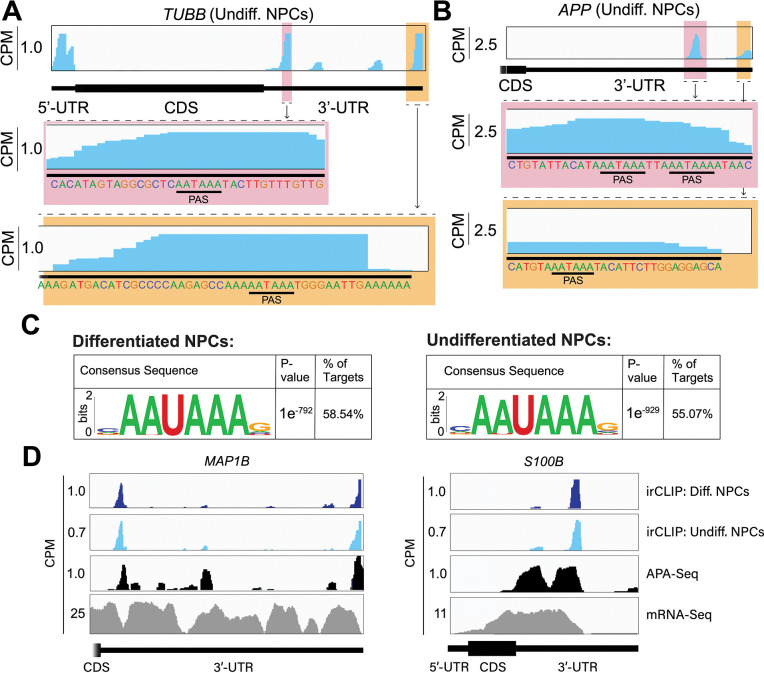
Crosslinking of eIF3 to mRNA 3’-UTRs adjacent to the poly(A) tail. **A)** Crosslinking of eIF3 across the *TUBB* mRNA in undifferentiated NPCs. A zoomed in view of *TUBB* mRNA 3’-UTR terminus with the eIF3 crosslinks is shown. The polyadenylation signal (PAS) is marked. **B)** Crosslinking of eIF3 across the *APP* mRNA 3’-UTR in undifferentiated NPCs. A zoomed in view of the two 3’-UTR regions to which eIF3 crosslinks are shown as well as their PAS sequences. **C)** Sequence logo of the eIF3 crosslinks located at 3’-UTRs in differentiated and undifferentiated NPCs. **D)** Crosslinking of eIF3, APA-Seq peaks, and mRNA-Seq across the *MAP1B* and *S100B* mRNAs in differentiated and undifferentiated NPCs.

In many instances, we observe eIF3 crosslinking peaks at the termini of annotated 3’-UTRs, adjacent to the poly(A) tail and where the polyadenylation signal (PAS; canonical PAS sequence = AAUAAA) is located (Figure 2A, *TUBB*). However, in addition to ends of annotated 3’-UTRs, we also observe the same type of crosslinking pattern across multiple regions within 3’-UTRs (Figure 1I, *VIM*). Looking at the nucleotide sequence of the regions within 3’-UTRs where eIF3 crosslinks, we observe that most of them contain PAS sequences as well (Figure 2B, *APP*). Hypergeometric optimization of motif enrichment (HOMER) motif discovery analysis^15^ of the eIF3 crosslinks that map to 3’-UTR regions revealed that in differentiated and undifferentiated NPCs 58% and 55% of the 3’-UTR eIF3 crosslinks, respectively, map to regions that are extremely enriched in the canonical PAS sequence AAUAAA (Figure 2C, Table S2, Table S3). We also find other enriched 3’-UTR motifs but none of them has either the level of eIF3 enrichment nor the statistical significance as AAUAAA (Figure S4). Overall, these data indicate eIF3 crosslinks near 3’-UTR termini of multiple isoforms, upstream of poly(A) tails in NPCs. In neurons, 3’-UTR isoforms are known to play key roles in mRNA localization and expression in different neuronal compartments^16,17^ and alternative polyadenylation is highly prevalent in the nervous system, leading to the expression of multiple mRNA isoforms with different 3’-UTR lengths^18^.

To test our hypothesis that the observed eIF3 crosslinking pattern reflects the expression of bona fide alternative 3’-UTRs in NPCs, we performed alternative polyadenylation next-generation sequencing (APA-Seq) in undifferentiated NPCs. Given that we observe similar eIF3 crosslinking levels in undifferentiated and differentiated NPCs, we did not perform APA-Seq in differentiated NPCs. APA-Seq creates sequencing libraries by amplifying the region upstream of a polyadenylation event, allowing the identification of all 3’-UTR mRNA isoforms being expressed for any given gene. Comparing our Quick-irCLIP and APA-Seq experiments, we observe that the eIF3 peaks located in 3’-UTRs map to the same location as APA peaks, indicating that the predominant eIF3 crosslinking peaks in 3’-UTRs identified by Quick-irCLIP localize to 3’-UTR termini, adjacent to the poly(A) tail. For instance, the eIF3 crosslinking peaks located in the 3’-UTR of the *MAP1B* mRNA correspond to APA-Seq peaks (Figure 2D). The aforementioned cases of *NES* and *VIM* mRNAs, where we observed eIF3 crosslinks in their 3’-UTRs, also have APA-Seq peaks at the same locations as the irCLIP peaks (Figure S5). As another example, our irCLIP experiment shows that eIF3 crosslinks to both the 5’- and 3’-UTRs of the *S100B* mRNA, which encodes for a calcium-binding protein highly expressed in brain cells and that is commonly used as a glial marker^19–21^. Within *S100B*’s 3’-UTR, we observe 2 major eIF3 crosslinking peaks at the 3’-UTR termini of 2 distinct *S100B* mRNA isoforms expressed in NPCs (Figure 2D). Interestingly, there are many instances where eIF3 crosslinks to 3’-UTR termini of multiple similarly expressed 3’-UTR isoforms to a different extent. For example, based on our APA-Seq data, there are two major *S100B* mRNAs with similar levels of expression that have different 3’-UTR ends. However, eIF3 preferentially crosslinks to the distal 3’-UTR isoform over the proximal 3’-UTR isoform (Figure 2D). Overall, these results indicate that the eIF3 crosslinking events that we observe in 3’-UTRs often map to 3’-UTR termini of multiple mRNA isoforms, upstream of poly(A) tails.

### eIF3 engages with 3’-UTR termini upon increased levels of protein synthesis

**Figure 3.**
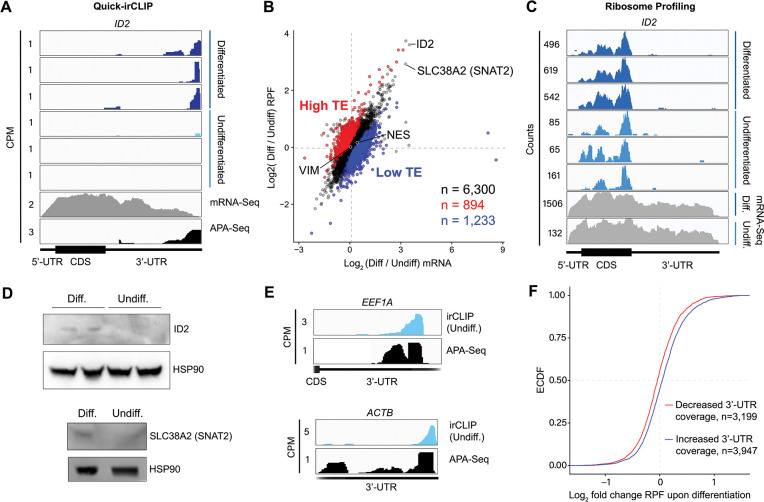
Ribosome profiling of undifferentiated and differentiated NPCs, and comparisons to eIF3 Quick irCLIP crosslinking. **A)** Crosslinking of eIF3 across the *ID2* mRNA in undifferentiated and differentiated NPCs. APA-Seq and mRNA-Seq data are also shown. **B)** log_2_ fold change of ribosomal footprints and log_2_ fold change of mRNA transcript levels upon NPC differentiation. Number of samples within each condition is shown. **C)** Ribosomal footprints across the *ID2* mRNA in undifferentiated and differentiated NPCs. mRNA-Seq data is also shown. **D)** Western blots of ID2, SLC38A2 (SNAT2) and Hsp90 in undifferentiated and differentiated NPCs. **E)** Crosslinking of eIF3 to the 3’-UTR of the *EEF1A* and *ACTB* mRNAs in undifferentiated NPCs. APA-Seq data is also shown. **F)** Cumulative distribution of the log_2_ fold change in ribosome occupancy after differentiated cells for genes found to have either an increase (blue) or decrease (red) in 3’-UTR irCLIP coverage upon differentiation, excluding genes with 3’-UTR less than 50 nts and without sufficient coverage to compute an adjusted p-value in both datasets.

Our Quick-irCLIP experiment showed that eIF3 interacts with the 3’-UTR termini of mRNAs expressed in both differentiated and undifferentiated NPCs. Interestingly, eIF3 interacts with the mRNA encoding the transcription regulator ID2 only upon NPC differentiation (Figure 3A). ID2 is involved in migrating NPC differentiation into olfactory dopaminergic neurons^22^. Therefore, it is possible that the observed eIF3 engagement at its 3’-UTR terminus in differentiated NPCs is due to *ID2*’s mRNA translation activation upon differentiation. This would suggest that eIF3 interactions with 3’-UTR termini are involved with active translation levels for a given transcript.

To test whether eIF3 engagement at 3’-UTR termini correlates with translation activity at a transcriptome-wide level, we performed ribosome profiling, a technique that is based on deep sequencing of ribosome-protected mRNA fragments (RPFs)^23^, in undifferentiated and differentiated NPCs. Here, we used a low-bias ribosome profiling approach that takes advantage of the enzymatic activities of reverse transcriptase (RT) from eukaryotic retroelements to reduce the technicalities associated with ribosome profiling^23,24^. In addition, this method uses P1 nuclease instead of the commonly used RNase I and replaces RNA ligation with Ordered Two-Template Relay (OTTR)^24^. P1 nuclease digestion not only preserves monosome-protected mRNA fragments, which are the commonly used fragments in ribosome profiling experiments, but also mRNA fragments protected by collided ribosomes (disomes), which report on ribosome stalling and provide a screenshot of ribosome quality control (RQC) pathways^24,26^.

Here, we deep sequenced monosome- and disome-protected fragments from undifferentiated and differentiated NPCs (Figure S6). We observe that about a third (monosome) and about a fifth (disome) of the libraries mapped to mRNAs (Figure S7A), similar to previous reports^24^. P1 nuclease digestion results in monosome footprints of 32 – 40 nts in length, observed in both differentiated and undifferentiated NPCs (Figure S7B). We also observe two populations of footprints in the disome library as previously reported upon P1 digestion, with the larger one corresponding to the “true disome” population (i.e., ≥ 60 nt disome footprints shown in Figure S7C), composed of colliding ribosomes and populations of two translating ribosomes in close proximity to each other, and to the “sub-disome” population (i.e., < 60 nt disome footprints shown in Figure S7C), proposed to correspond to 80S monosomes in close proximity to a scanning 40S ribosomal subunit during translation initiation or to a 40S ribosomal subunit from which a 60S ribosomal subunit was recently dissociated from during translation termination^24^. In agreement with the translational burst that we identified (Figure 1C), our monosome profiles show an increase in RPFs around the start codon in differentiated NPCs, indicating an increase of initiating ribosomes in differentiated NPCs with respect to the undifferentiated conditions (Figure S7D). We observed a global increase in ribosome traffic after differentiation as evidenced by the increase in true-disome footprints among CDS and terminating codons (Figure S7C). By DESeq2 analysis, which does not consider transcript length in read-count normalization, we found in both differentiated and undifferentiated NPCs an increase in true-disome CDS occupancy, relative to monosome, correlates with CDS length (Figure S7E). This was expected since longer coding sequences are more likely to have more translating ribosomes per transcript than shorter ones.

Our ribosome profiling experiment indicates that for most mRNAs (n = 6,300 of a total of 8,427, i.e. ~75%), translation efficiency levels (RPF counts / mRNA counts) remain unchanged upon NPC differentiation (Figure 3B). *ID2* is one of the transcripts with the highest increase in both RPFs and mRNA counts upon NPC differentiation (Figure 3B). Specifically, we observe a >5-fold RPF count increase upon NPC differentiation (Figure 3C), indicating a significant burst in *ID2* mRNA translation activity. However, we also observe that NPC differentiation results in a significant increase in *ID2* mRNA transcript levels (~ 15-fold, Figure 3C). Western blots of ID2 and a second protein SLC38A2 (SNAT2) with increased RPFs on its mRNA in differentiated vs. undifferentiated NPCs are in agreement with the ribosome profiling data (Figure 3D). Notably, this observed step-wise increase in *ID2* mRNA levels and translation is well corroborated by our observed increase in eIF3 crosslinking to *ID2* mRNA’s 3’-UTR termini, suggesting a connection for eIF3 in maintaining and promoting *ID2* translation upon differentiation beyond the 5’-UTR.

We next looked at the most actively translated transcripts in NPCs by sorting the ribosome profiling hits based on their amount of RPFs. We noticed that the most actively translated transcripts, such as *EEF1A* and *ACTB*, also present significant eIF3 peaks at their 3’-UTR termini (Figure 3E). Together with the *ID2* mRNA results, these data suggest that eIF3 interacts with 3’-UTR ends of mRNAs being actively translated.

To test whether the observed increase in eIF3 crosslinking to 3’-UTRs was linked to an increase in ribosome translation at a global level, we performed DESeq2 on the counted 3’-UTR eIF3 irCLIP reads to genes with 3’-UTRs longer than 50 nts and sufficient coverage to compute a false discovery rate adjusted p-value^27^, and defined each gene as having either more (blue) or less (red) 3’-UTR crosslinking upon differentiation. Indeed, we found genes which had an increase in 3’-UTR irCLIP crosslinking upon differentiation tended to have an increase in ribosome occupancy (Figure 3F). Overall, our ribosome profiling data combined with our eIF3 irCLIP data suggest that, upon NPC differentiation, there is a global increase in eIF3 engagement at 3’-UTR termini that correlates with an overall increase in protein synthesis.

### eIF3 interactions with 3’-UTR termini requires polyadenylation but is independent of poly(A) binding proteins

**Figure 4.**
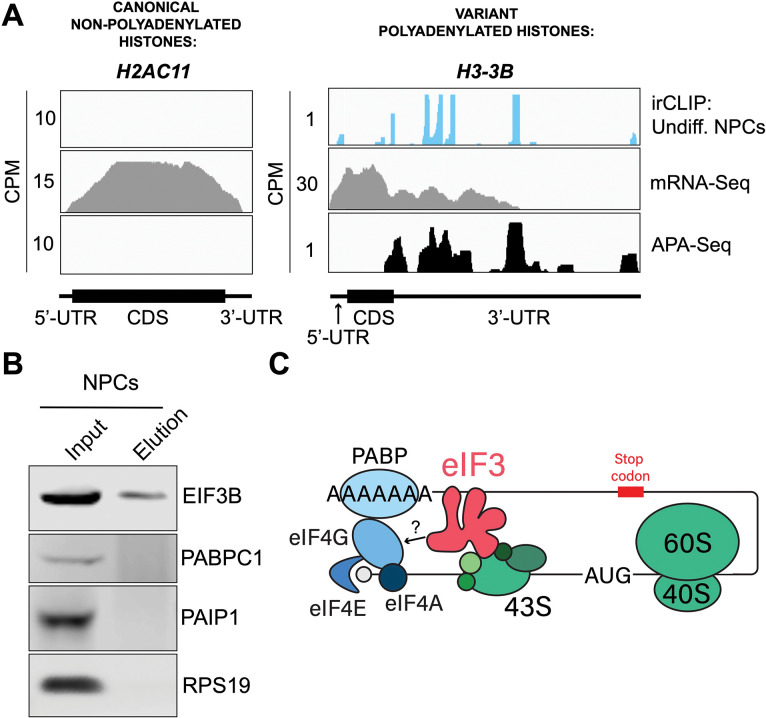
Crosslinking of eIF3 to polyadenylated mRNAs and model for mRNA circularization. **A)** Crosslinking of eIF3 across the canonical non-polyadenylated histone *H2AC11* mRNA and the variant polyadenylated histone *H3–3B* mRNA in undifferentiated NPCs. APA-Seq and mRNA-Seq data for these regions are also shown. **B)** Western blots of EIF3B immunoprecipitations from undifferentiated NPCs. **C)** Model for eIF3 contribution to mRNA circularization in highly translated transcripts.

eIF3 crosslinking at 3’-UTR termini suggests that it must be in close proximity to the poly(A) tail and poly(A) tail binding proteins (PABPs). To determine whether the presence of eIF3 at 3’-UTR ends requires polyadenylation, we looked at histone mRNAs. Canonical histone mRNAs are the only known cellular non-polyadenylated mRNAs in eukaryotes due to their cell-cycle dependency and their need to be degraded rapidly. On the other hand, several variant histone mRNAs are not cell-cycle dependent and are polyadenylated. In non-polyadenylated canonical histone mRNAs (*H2AC1*, *H3C1*, and *H4–16*), we see no traces of eIF3 crosslinking at 3’-UTR termini nor APA events, as expected (Figure 4A, Figure S8A). However, for the variant polyadenylated histone mRNAs (*H3–3B* and *H2AZ1*), we observe eIF3 crosslinks that localize with APA-Seq peaks (Figure 4A, Figure S8A). These data indicate that eIF3 only interacts with 3’-UTR termini of mRNAs that are polyadenylated, and thus should be in close proximity to the poly(A) tail.

Our observations that eIF3 interacts with 3’-UTR termini in highly translated mRNAs support a model in which these mRNAs are circularized and their 5’ and 3’ ends are in close proximity. Since we observe eIF3 crosslinks adjacent to the poly(A) tail, it is conceivable that this eIF3-mediated mRNA circularization is stabilized through interactions between eIF3 and poly(A)-binding proteins. eIF3 has been reported to interact with the poly(A)-binding protein interacting protein 1 (PAIP1) in HeLa cells through the EIF3G subunit to promote closed loop formation^28^. As in our Quick-irCLIP experiment, we immunoprecipitated assembled eIF3 complexes using an anti-EIF3B antibody from UV-crosslinked undifferentiated NPCs and immunoblotted against PAIP1 and against the major cytoplasmic isoform of the PABPs (PABPC1). Our results indicate that despite eIF3 being in close proximity to the location of the PABPs, these do not elute with it (Figure 4B). Immunoprecipitation of eIF3 complexes from UV-crosslinked HEK293T gave the same results (Figure S8B). It is possible that the eIF3 interactions with poly(A)-binding proteins are transient and eIF3 immunoprecipitation results in the disruption of these complexes. Therefore, to maintain the integrity of these potentially transient interactions, we treated NPCs with the protein-protein crosslinker dithiobis(succinimidyl propionate) (DSP) before cell collection and eIF3 immunoprecipitation. DSP is a thiol-cleavable crosslinker so eluates can be treated with dithiothreitol (DTT) to cleave the disulfide bond in the spacer arm of DSP as a way to separate eIF3 from its polypeptide interactors. Our results show that even when protein-protein transient interactions are covalently linked by DSP treatment, PABPC1 and PAIP1 do not elute with eIF3 in NPCs (Figure S8C). The same experiments performed in HEK293T gave the same results (Figure S8C). Overall, these results suggest that eIF3 engagement with 3’-UTR termini in highly translated transcripts requires polyadenylation but is independent of interactions with poly(A)-binding proteins.

## DISCUSSION

Here, we identified the transcripts that interact with eIF3 in hPSC-derived NPCs. NPCs that have undergone differentiation toward forebrain neurons exhibit a global increase in protein synthesis that we identified to occur 2 hours post differentiation treatment. Our Quick-irCLIP experiment shows that eIF3 predominantly crosslinks to 3’-UTRs in NPCs. By performing APA-Seq, we discovered that these eIF3 3’-UTR crosslinks map to 3’-UTR termini of multiple mRNA isoforms, adjacent to poly(A) tails and where the polyadenylation signal is located. Interestingly, we have also identified eIF3 irCLIP peaks within mRNA coding regions such as in *NES* mRNA (Figure S5B) that colocalize with APA-Seq peaks, indicating that different nestin protein isoforms are expressed in NPCs. Therefore, in addition to providing mRNA isoform-specific expression information, our APA-Seq experiment could potentially be used to investigate the expression of specific protein isoforms in NPCs.

Here, we also observe that early NPC differentiation results in eIF3 engagement with the 3’-UTR terminus of the mRNA encoding the transcription regulator ID2. By ribosome profiling, we determined that this increase in eIF3 engagement on *ID2* mRNA is correlated with an increase in the levels of active translation of ID2 (Figure 3). At a global level, we also observe that differentiated NPCs present higher levels of eIF3 crosslinks to 3’-UTRs and higher levels of active translation with respect to undifferentiated NPCs (Figure 3F), suggesting that these two phenomena are correlated. In addition, we observe that the highest levels of eIF3 crosslinking at 3’-UTR ends are observed on the most actively translated mRNAs (e.g. *EEF1A* and *ACTB*, Figure 3E). Overall, these data suggest that actively translated mRNAs engage eIF3 at their 3’-UTR termini, although the underlying molecular mechanisms of the eIF3 engagement with the 3’-UTR terminus remain to be determined.

Our APA-Seq experiment performed in NPCs maps all polyadenylation events and it infers mRNA expression at isoform level. We found several instances where eIF3 CLIP peaks that colocalize with polyadenylation events have different peak heights with respect to those of the APA-Seq experiment. For instance, our APA-Seq data suggests that the proximal and distal 3’-UTR isoforms of the *S100B* mRNA are expressed at similar levels in NPCs (Figure 2D). However, we observe higher eIF3 engagement levels with the distal isoform than with the proximal isoform. Given that our ribosome profiling experiment indicates eIF3 engagement at 3’-UTR termini is observed on actively translated mRNAs, our eIF3 irCLIP and APA-Seq datasets could potentially be used in combination to determine active translation levels at mRNA isoform level, a feature that cannot be determined by ribosome profiling alone since RPF counts cannot be mapped to multiple mRNA isoforms.

eIF3 is an initiation factor that is usually associated with 5’-UTRs, where it organizes interactions between other initiation factors and the 40S ribosomal subunit during translation initiation. Its presence at the very end of 3’-UTRs of actively translated mRNAs supports the mRNA circularization model, where the 5’ and 3’ ends of highly translated mRNAs are proposed to be brought in close proximity to each other by the interactions of multiple proteins that bridge 5’-UTRs with 3’-UTRs^29^. It is possible that our observation of eIF3 at 3’-UTR termini is due to the recycling of eIF3 from 3’-UTRs to 5’-UTRs for successive rounds of translation. However, this would require eIF3 association with translating ribosomes until translation termination and subsequently scanning through the entire length of 3’-UTRs either by direct interactions with mRNAs or through binding with post-termination 40S ribosomal subunits scanning through 3’-UTRs until the poly(A) tail is encountered. If that were the case one would expect to observe higher eIF3 levels at the ends of short 3’-UTRs than at those of long 3’-UTRs, and we observe no evidence of this (Figure S9). Furthermore, recent results indicate that eIF3 associates with elongating ribosomes but it dissociates after ~ 60 codons from the start codon, before termination^23^. Alternatively, given the large size of eIF3 (800 KDa in humans), it is possible that during mRNA circularization eIF3 interactions with 3’-UTR termini are accomplished by a specific eIF3 module while performing its canonical role as a scaffold protein during translation initiation (Figure 4C). In the closed loop model of translation it is proposed that the 5’ and 3’ ends of translated mRNAs are connected via the multiprotein-RNA interactions composed of the mRNA 5’ cap - eIF4E - eIF4G - PABP - poly(A) tail^29^, which are conserved across phylogeny. The disruption of these interactions results in a decrease in translation^30–33^. mRNA circularization promoted by the canonical closed loop model has been observed *in vitro* by atomic force microscopy and remains the only structural evidence for the model to date^29^. Since then, additional interactions between proteins regularly associated with 5’-UTRs and proteins commonly associated with 3’-UTRs have been discovered. For example, EIF3H has been shown to interact with METTL3, which binds to *N*6-methyladenosine (m6A) modified sites near the stop codon, an interaction proposed to enhance translation, indicating a role in looping highly translated mRNAs^34^. Another eIF3 subunit, EIF3G, has also been reported to be involved in mRNA circularization. Studies performed in HeLa cells showed EIF3G coimmunoprecipitates with PAIP1, suggesting a role in bridging 5’-UTRs with poly(A) tails^28^. Here, we immunoprecipitated the eIF3 complex from NPCs and HEK293T using an anti-EIF3B antibody but did not observe PAIP1 nor other poly(A)-associated proteins in the eIF3 eluates. It is possible that these divergent results are due to the fact that HeLa extracts present high levels of EIF2α phosphorylation, which are not observed in other cell extracts such as HEK293T^36^, suggesting that protein translation is highly dysregulated in HeLa cells and therefore the mechanisms of translation regulation might be significantly different than in other cells. Furthermore, the EIF3G-PAIP1 interaction is reported to be RNA independent, while our observations reveal an interaction of eIF3 with 3’-UTRs of polyadenylated mRNAs, so it is also possible that these are two independent mechanisms.

It is worth noting that the eIF3 3’-UTR termini crosslinking pattern was not observed in the two previous studies that used PAR-CLIP to identify the eIF3 mRNA-binding sites in HEK293T cells^9^ and Jurkat cells^11^. Quick-irCLIP uses a short UV wavelength (254 nm) to crosslink RNA to proteins, whereas PAR-CLIP is performed at 365 nm and requires the cellular internalization of 4-thiouridine (4SU). eIF3 is a large multisubunit complex so it is possible that different UV wavelengths capture transcript interactions through different eIF3 modules containing different amino acid propensity to crosslink to mRNAs at the two wavelengths. Using a different UV crosslinking method with respect to the previous work performed in HEK293T and Jurkat is a caveat when it comes to comparative studies such the one reported on Figure S1, in which eIF3-crosslinked mRNAs in NPCs are nearly disjoint from those crosslinked to eIF3 in HEK293T and Jurkat cells. However, it is striking that we were still able to identify some common 5’-UTR eIF3 binding sites (Figure S2).

Overall, our results provide new insights into the repertoire of roles that eIF3 plays during translation initiation. Besides its canonical role as a scaffold protein for initiation complex assembly^7^ and its non-canonical roles in translation activation/repression by direct mRNA interactions at 5’-^9^ and 3’-UTRs^11^, our study reveals a role in which eIF3 interacts with 3’-UTR termini of highly translated mRNAs and suggests eIF3 assists in the communication between 5’ and 3’ mRNA ends, supporting the mRNA circularization model to promote efficient recycling of ribosomes and translation factors for successive rounds of translation.

## MATERIALS AND METHODS

### Maintenance and propagation of human pluripotent stem cells (hPSCs)

The surface of all cultureware used for the maintenance and propagation of human pluripotent stem cells (WTC-11 line) was coated with Corning Matrigel (Fisher Scientific) prior to cell seeding. hPSCs were maintained under feeder-free conditions in complete mTeSR Plus medium (Stem Cell Technologies) and passaged using Gentle Cell Dissociation Reagent (Stem Cell Technologies).

### Generation of hPSC-derived neural progenitor cells (NPCs)

NPCs were generated from hPSCs using the embryoid body protocol (Stem Cell Technologies) following the manufacturer’s protocol. At day 17, hPSC-generated NPCs (passage 1) were maintained and propagated using complete STEMdiff^™^ Neural Progenitor Medium (Stem Cell Technologies). Cells from passages 1 to 6 were cryopreserved.

### Maintenance and propagation of hPSC-derived NPCs

The surface of all cultureware used for the maintenance and propagation of hPSC-derived NPCs was coated with Corning Matrigel (Fisher Scientific) prior to cell seeding. Maintenance and propagation of hPSC-derived NPCs was performed following manufacturer’s instructions. Briefly, NPCs were maintained using complete STEMdiff^™^ Neural Progenitor Medium (Stem Cell Technologies) for 7–9 days, with medium change every other day. NPCs were passaged using Accutase (Stem Cell Technologies) and seeded onto Matrigel-coated cultureware at an initial concentration of 1.25×10^5^ cells/mL.

### Differentiation treatment of hPSC-derived NPCs and identification of the global increase in protein synthesis

For differentiation studies, hPSC-derived NPCs were seeded at an initial concentration of 5×10^5^ cells/mL in STEMdiff^™^ Neural Progenitor Medium (Stem Cell Technologies) and allowed to sit at 37 °C for 48 hrs. After 48 hrs, the medium was changed to complete STEMdiff^™^ Forebrain Neuron Differentiation medium (Stem Cell Technologies) (for differentiated NPCs) or to fresh complete STEMdiff^™^ Neural Progenitor Medium (for undifferentiated NPCs) for 2 hrs at 37 °C and collected by washing with 1 mL PBS and scraping, followed by brief centrifugation. Cells were stored at −80 °C until further use. For protein synthesis assays, cells were treated with 20 mM puromycin for 15 min at 37 °C prior to collecting.

### eIF3 immunoprecipitation

Fifty μL of slurry Dynabeads Protein G (Invitrogen) were washed twice with 1 mL PBS + 0.01% Tween 20. Then, 25 μL of anti-EIF3B antibody (Bethyl, A301–757A) were added to washed beads and incubated at room temperature for 40 min with mixing by gentle rotation. For IPs with anti-EIF3B antibody performed to look for potential interactions with polyA-binding proteins, the anti-EIF3B antibody was crosslinked to Dynabeads Protein G with (bis(sulfosuccinimidyl)suberate) (BS3) (ThermoFisher Scientific) following the manufacturer’s protocol. For cell lysis, NPCs were lysed using lysis buffer (50 mM Hepes-KOH, pH 7.5, 150 mM KCl, 5 mM MgCl_2_, 2 mM EDTA, 0.5% EDTA, 0.5 mM DTT, 1 Complete EDTA-free Proteinase Inhibitor Cocktail tablet per 10 mL of buffer). For IPs performed to look for potential EIF3B transient interactions with polyA-binding proteins, prior to collection, NPCs and HEK293T were treated with the protein-protein crosslinker dithiobis[succinimidylpropionate] (DSP) (1 mM) in PBS for 30 min at room temperature, and reaction was quenched by the addition of 10 mM Tris-HCl, pH 7.5 for 15 min at room temperature. Cells were then collected and lysed as described above. The cell pellets resuspended in lysis buffer were incubated on ice for 10 min, passed through a 18G needle four times, and centrifuged at 13,000*g* for 10 min at 4 °C. Twenty μL of lysate was saved as 5% input and the remaining lysate was loaded to washed beads conjugated to anti-eIF3b antibody and incubated at 4 °C for 2 hrs with gentle rotation. Next, the beads were washed three times with lysis buffer. Then, eIF3-bound RNAs were eluted from the beads with 50 μL 1X NuPAGE LDS sample buffer and boiled at 70 °C for 5 min. Samples were loaded onto a 4–12% Bis-Tris gel (Invitrogen), transferred to a nitrocellulose membrane, which was blotted against the desired proteins.

### Quick-irCLIP

Quick-irCLIP was performed as previously described^13^. NPCs subjected to Quick-irCLIP were prepared as follows. NPCs were seeded at an initial concentration of 5×10^5^ cells/mL and incubated at 37 °C for 48 hrs in STEMdiff^™^ Neural Progenitor Medium. Then, the medium was changed to either fresh STEMdiff^™^ Neural Progenitor Medium (undifferentiated cells) or STEMdiff^™^ Forebrain Neuron Differentiation medium (differentiated NPCs) and incubated at 37 °C for 2 hrs. Cells were then crosslinked by irradiating them on ice with 0.15 J/cm^2^ of 254 nm UV light. Then, cells were collected by scraping in 1 mL PBS followed by centrifugation at 150× *g* for 5 min and stored at −80 °C until further use. Next, NPC pellets were allowed to thaw on ice for 10 min. In the meantime, 100 μL of slurry Dynabeads (Invitrogen) were washed twice with 1X PBS + 0.01% Tween 20. Then, 25 μL of anti-EIF3B antibody (Bethyl, A301–757A) were added to the washed beads solution and incubated at room temperature for 40 min with gentle rotation. Then, thawed NPCs were lysed by the addition of 1 mL of lysis buffer (50 mM Hepes-KOH, pH 7.5, 150 mM KCl, 5 mM MgCl_2_, 2 mM EDTA, 0.5% EDTA, 0.5 mM DTT, 1 Complete EDTA-free Proteinase Inhibitor Cocktail tablet per 10 mL of buffer) and allowed to sit on ice for 10 min. The resuspended pellet was then passed through a 18G needle four times, and centrifuged at 13,000× *g* for 10 min at 4 °C. Lysate protein concentration was determined by Bradford assay to determine the amount needed to obtain 0.5 mg of protein. Then, lysate with 0.5 mg per sample was transferred to a new Eppendorf tube. Ten μL of RNase I (Fisher Scientific) (1:1,000 dilution) and 2 μL of Turbo DNase (Fisher Scientific) were added to the lysate and allowed to mix at 37 °C for 3 minutes while shaking at 1100 rpm. Samples were then immediately placed on ice for 3 min followed by centrifugation at 18,000× *g* for 10 min at 4 °C. Supernatants were then transferred to new tubes. At this point, washed beads conjugated to anti-EIF3B antibody were washed twice with 1X PBS + 0.01% Tween 20 and washed once with lysis buffer. Then, RNAse-treated lysates were loaded to conjugated beads and incubated at 4 °C for 2 hrs with gentle rotation. Beads were then washed twice with high-salt wash buffer (50 mM Hepes-KOH, pH 7.5, 500 mM KCl, 0.5% Nonidet P-40 alternative, 0.5 mM DTT, 5 mM MgCl_2_, 1 Complete EDTA-free Proteinase Inhibitor Cocktail tablet per 10 mL of buffer), and then with PNK wash buffer (20 mM Tris-HCl, pH 7.5, 10 mM MgCl_2_, 0.2% Tween-20). Beads were subsequently resuspended in 20 μL dephosphorylation reaction mix (1X PNK buffer, pH 6.5, 5 units of T4 polynucleotide kinase (New England Biolabs), 0.5 μL RNaseOut Ribonuclease inhibitor (Fisher Scientific)) and incubated for 20 min at 37 °C while shaking at 1100 rpm. Next, beads were washed once with PNK buffer, then washed once with high-salt wash buffer, and then washed again once with PNK buffer.

The following steps describe the ligation of the adaptor previously conjugated to an IR dye. Briefly, 20 μL of ligation reaction mix was prepared (1X ligation buffer, 5 units of T4 RNA ligase I (New England Biolabs), 0.5 μL RNaseOut Ribonuclease inhibitor, 1.5 μL of 10 mM infrared adaptor oligonucleotide, 4 μL polyethylene glycol 400). PNK buffer was removed from the beads and then 20 μL of ligation reaction mix were added to the beads and allowed to incubate at 16 °C while shaking at 1100 rpm overnight.

Next, beads were washed once with PNK buffer, twice with high-salt wash buffer, and once more with PNK buffer. Beads were then transferred to new Eppendorf tubes and resuspended in PNK buffer. Meanwhile, 500 mL of 1X NuPAGE MOPS-SDS buffer were prepared and 500 μL antioxidant (Fisher Scientific, cat#: NP0005) were added fresh before loading buffer use. PNK buffer was then removed from beads and samples were resuspended with 13 μL nuclease-free water, 2 μL sample reducing agent (Fisher Scientific, cat#: NP0004), and 5 μL 4X NuPAGE protein loading buffer. Samples were then incubated at 80 °C for 5 min, briefly centrifuged, and loaded onto a 4–12% SDS NuPAGE Bis-tris gel. The gel was run for 50 min at 180 V. Protein-RNA complexes were then transferred to a nitrocellulose membrane for 90 min at 30 V and then were visualized using a near infrared imager (Li-COR Odyssey CLx). The membrane was then put in a light-protected box. A grayscale image of the membrane was printed on acetate film, the membrane was wrapped in a plastic wrap and then the membrane and grayscale image were aligned using the ladder as reference.

Using a razor blade per sample, a box corresponding to the molecular weight range of eIF3 subunits EIF3A, EIF3B, EIF3C, and EIF3D (140 to 65 KDa) was cut into small pieces and transferred to a new Eppendorf tube. Proteins in the cut box were then digested by adding 10 μL Proteinase K (20 ug/μL) and 200 μL PK buffer (100 mM Tris-HCl, pH 7.4, 50 mM NaCl, 10 mM EDTA) and incubated at 37 °C for 20 min while shaking at 1100 rpm. Then, 200 μL PK-Urea buffer (100 mM Tris-HCl, pH 7.4, 50 mM NaCl, 10 mM EDTA, 7 M urea) and incubated for an additional 20 min while shaking at 37 °C at 1100 rpm. Supernatants were then transferred to phase lock heavy columns along with 400 μL neutral phenol-chloroform and incubated for 5 min at 30 °C while shaking at 1100 rpm. Samples were then centrifuged for 5 min at 18,000× *g* at room temperature. The aqueous upper layers were transferred to new low-binding 1.5 mL tubes and centrifuged for 1 min at 18,000× *g* at room temperature. Samples were then transferred to new low-binding 1.5 mL tubes and 0.75 μL glycogen (RNA grade, Fisher Scientific, cat#: FERR0551), 40 μL 3 M sodium acetate (pH 5.5), and 1 mL 100% ethanol were added per sample. Samples were briefly vortexed and precipitated overnight at −20 °C.

The next day, samples were centrifuged for 20 min at 18,000× *g* at 4 °C, supernatants removed, leaving approximately 50 μL of the RNA pellet. Then, 1 mL of 80% ethanol was added, without overly agitating the pellet. Supernatants were carefully removed and pellets were air-dried at room temperature. Pellets were then resuspended in 7 μL nuclease-free water and transferred to new PCR tubes. Sequencing libraries were created following the protocol described in the SMARTer^®^ smRNA-Seq Kit for Illumina user manual (Takara Bio).

### Total RNA cDNA library preparation

RNA samples were extracted from differentiated NPCs or undifferentiated NPCs treated for 2 hrs with STEMdiff^™^ Forebrain Neuron Differentiation medium (Stem Cell Technologies) for differentiated NPCs, or with fresh complete STEMdiff^™^ Neural Progenitor Medium for undifferentiated NPCs, using Trizol reagent (Thermo Fisher). cDNA libraries were prepared using NEBNext^®^ ultra II Directional RNA Library Prep Kit for Illumina (NEBNext rRNA Depletion Kit v2).

### Quick-irCLIP Computational Analysis

Quick-irCLIP and Total RNA cDNA libraries were sequenced on an Illumina NovaSeq S1 150PE platform. Cutadapt^37^ (version 3.5), with a minimum length of 20 nucleotides, was used to remove 3’ adapter sequences. Strand-specific alignments were generated using HISAT^40^ v.2.2.1 and hg38. Duplicate reads were removed with Picard (v2.21.9, https://broadinstitute.github.io/picard/) MarkDuplicates, followed by peak calling with HOMER^15^ (v4.11) findPeaks and a FDR cutoff of 0.01. Bedtools intersect (v2.29.2) was used to identify statistically significant peaks (BH-adjusted p-value < 1.0e-04) present in all replicates, then motifs were generated with HOMER findMotifsGenome. Peak statistics were tabulated using custom Python scripts. For coverage graphs, bams were CPM (Counts Per Million) normalized and converted to BigBiwgs with Deeptools^38^ (v3.5.1) then visualized with IGV. Figures without irCLIP and APA-seq replicate tracks represent the union of all replicate reads, prepared with Samtools^39^ (v1.17) merge and CPM normalization.

### Comparison of eIF3 Quick-irCLIP results in NPCs with eIF3 PAR-CLIP results in Jurkat and HEK293T cells

Since the eIF3 PAR-CLIP experiments done in Jurkat and HEK293T cells were performed to identify the transcripts that interact with individual eIF3 subunits, first we obtained the RNAs that are common interactors of eIF3 subunits EIF3A (328 RNA-EIF3A reported clusters), EIF3B (264 RNA-EIF3B reported clusters), and EIF3D (356 RNA-EIF3D reported clusters) in HEK293T^9^. The reason we only picked these subunits for this comparison study is because in our Quick-irCLIP experiment we only isolated the RNAs bound to these subunits. This rendered a list of 175 RNA-eIF3 common clusters between subunits EIF3A, EIF3B, and EIF3D in HEK293T. We then picked the top 400 transcripts that interact with activated Jurkat cells^11^ and performed the same analysis to find the common RNAs that bind to subunits EIF3A, EIF3B, and EIF3D, giving a total of 209 transcripts. Next, we identified the common transcripts between the two studies by comparing the 175 common in HEK293T with the 209 common in activated Jurkat cells, giving a total of 27 transcripts. Finally, we picked the top 210 transcripts (common in the 3 replicates) identified by eIF3 Quick-irCLIP in differentiated NPCs and compared them to the 175 common RNAs in HEK293T cells (resulting in 8 NPC-HEK293T common RNAs) and to the 209 common RNAs in activated Jurkat cells (resulting in 12 NPC-Jurkat common RNAs). Performing the same analysis with the top 210 common transcripts that bind eIF3 in undifferentiated NPCs gave the same results.

### Alternative Polyadenylation (APA)-Seq

Total RNA was extracted using Trizol Reagent from NPCs that were seeded at an initial concentration of 5×10^5^ cells/mL and incubated at 37 °C for 48 hrs in STEMdiff^™^ Neural Progenitor Medium. After 48 hrs, the media was changed to fresh STEMdiff^™^ Neural Progenitor Medium and NPCs were incubated 37 °C for 2 hrs and collected. Total RNA was then extracted (Trizol reagent) from NPCs. 3’ mRNA-Seq libraries were prepared from 500 ng total RNA using QuantSeq REV kits (Lexogen) according to the manufacturer’s protocol.

### APA-Seq Computational Analysis

APA-Seq and Total RNA cDNA libraries were sequenced on an Illumina NovaSeq S1 150PE platform. Cutadapt^37^ (version 3.5) with a minimum length of 20 nucleotides was used to remove 3’ adapter sequences. RNA-seq reads were aligned using Hisat2^40^ v.2.2.1 and mapped to the hg38 reference genome. SAMtools^39,41^ was used to remove PCR duplicates and filter uniquely mapped reads.

### Ribosome profiling

#### Preparation of NPC lysates:

Ribosome profiling was performed as previously described^24,42^, with the following modifications. Briefly, NPCs were seeded in four Matrigel-coated 15-cm dishes per condition and allowed to reach 70–80% confluency in STEMdiff^™^ Neural Progenitor Medium (Stem Cell Technologies). Then, they were treated for 2 hrs with STEMdiff^™^ Forebrain Neuron Differentiation medium for differentiated NPCs, or STEMdiff^™^ Neural Progenitor Medium for undifferentiated NPCs. NPCs were treated with 100 μg/mL cycloheximide for 5 min prior to collection and washed with ice-cold PBS containing 100 μg/mL cycloheximide. PBS was discarded and 1.2 mL Lysis Buffer (20 mM Tris-HCl, pH 7.4, 150 mM NaCl, 5 mM MgCl2, 1 mM DTT, 100 μg/mL cycloheximide, 1 % v/v Triton X-100, 25 U/mL Turbo DNase I) was added and cells were scraped, transferred to an Eppendorf tube, and allowed to sit on ice for 15 min. Cells were then passed 10 times through a 26 gauge needle and lysate was clarified by centrifugation (10 min, 20,000× *g*, 4 °C). Supernatants were recovered and stored at −80 °C.

#### RNA quantification of NPC lysates:

RNA concentration of lysates diluted in 1X TE buffer was quantified by using Quant-iT RiboGreen RNA kit (ThermoFisher) through direct comparison with a 0.0 – 1.0 ng/μL rRNA standard curve (ThermoFisher).

#### P1 nuclease treatment of NPC lysates and ribosome pelleting:

The pH of lysates was adjusted to 6.5 for optimal P1 nuclease activity by adding RNase-free 300 mM Bis-Tris pH 6.0 per 100 μL of cell lysate. Then, 450 Units of P1 Nuclease (100 U/μL, New England Biolabs) were added per 30 μg of RNA and lysates were incubated at 37 °C for 1 hr with gentle rotation. In the meantime, sucrose density gradients were prepared by dissolving 1M D-sucrose in 10 mL Polysome Buffer (20 mM Tris-HCl, pH 7.4, 150 mM NaCl, 5 mM MgCl_2_, 1 mM DTT, 100 μg/mL cycloheximide) and 900 μL were added to 13 × 56 mm polycarbonate thick wall tube (Beckman Coulter). Then, 300 μL of digested lysates were carefully added to the top of the sucrose gradient and centrifuged at 100,000 RPM for 1 hr at 4 °C in a pre-chilled 4 °C TLA 100.3 rotor. The sucrose cushion was then aspirated and 30 μL nuclease-free water were added to the ribosome pellet, allowing it to sit on ice for 10 min. The ribosome pellet was disrupted by pipetting and 300 μL TRIzol (ThermoFisher) were added and the entire volume was transferred to a pre-chilled tube, vigorously vortexed and stored at −80 °C.

#### mirRICH small RNA enrichment from pelleted ribosomes:

A modified TRIzol (ThermoFisher) RNA extraction was performed by first adding chloroform, vortexing, centrifuging, and isolating the aqueous phase as described by the manufacturer. RNA was precipitated by adding 100% isopropanol and incubating on ice for 15 min. RNA was pelleted by centrifugation at max speed for 15 min at 4 °C. Supernatant was carefully removed and pellets were allowed to air-dry at room temperature for 2 hrs. Dried pellets were then resuspended in 10 μL nuclease-free water, vortexed, and briefly centrifuged and incubated at room temperature for 5 min. Resuspended pellets were vortexed and briefly centrifuged again and 9 μL of the eluate liquid was removed and purified by RNA & Clean Concentrator-5, eluted in 25 μL nuclease-free water, and quantified by Nanodrop.

#### cDNA size selection, elution, and quantification:

The protocol from Ferguson et al.^24^ was followed for the OTTR reaction. OTTR reaction products were resuspended in bromophenol blue formamide loading dye. OTTR cDNAs were resolved by electrophoresis in 0.6X TBE 8% Urea-PAGE and visualized by fluorescence using the 5’ Cy5-dye. Gel excision and elution was performed as described^24^. To quantify cDNA by qPCR and determine the number of PCR cycles needed for library amplification, we followed a previously described method^42^.

#### Ribosome profiling computational analysis:

Ribosome profiling and Total RNA cDNA libraries were sequenced on an Illumina NovaSeq 150PE platform. The adapters were trimmed, the unique molecular identifier (UMI) was appended to the FASTQ entry header, and reads shorter than 20 were excluded using Cutadapt, as described^24^:

cutadapt -m 2 -a GATCGGAAGAGCACACGTCTGAACTCCAGTCAC $tmpvar.fastq |

cutadapt -u 7 --rename=‘{id} UMI={cut_prefix}’ - | cutadapt -u −1 --

rename=‘{id}_{comment}_primer={cut_suffix}’ - | cutadapt -m 20 -q 10 –

Human references described here were used as in ^24^. Briefly, human rRNA sequences NR_023379.1, NR_146151.1, NR_146144.1, NR_145819.1, NR_146117.1, NR_003285.3, NR_003286.4, X12811.1, and NR_003287.4 from NCBI and ENST00000389680.2 and ENST00000387347.2 from Ensembl were used as the rRNA reference. The human tRNA sequences and references were prepared as described^43^. ncRNA from Ensembl and lncRNA from Gencode were concatenated. The primary assembly of the human gDNA from NCBI, GRCh38, was used. 18,640 NCBI RefSeq MANE v0.95 transcripts were used, and the 5ʹ-UTR, CDS, and 3ʹ-UTR lengths were parsed from the GenBank entries using a custom script. The 13 mitochondrial mRNA sequences from Ensembl were also included in the mRNA reference, but typically excluded from analysis.

Contaminating reads were removed with a sequential analysis pipeline as described^24^. Briefly, the trimmed reads were first mapped to the rRNA reference (bowtie -v 3 -a --best --norc), then tRNA reference by tRAX^43^, then ncRNA reference (bowtie -v 3 -a --best --norc), and then mtDNA reference (bowtie -v 2 -a --best --norc). The remaining reads were then mapped to the mRNA references (bowtie -v 2 -m 200 -a --norc) and sorted (samtools sort -n). Remaining reads were aligned to the gDNA reference (bowtie -v 2 -a –best) before mapping unaligned reads to a reference of size-selecting oligos and adapter oligos (bowtie -v 2 -a --best).

To measure CDS occupancy we used Bedtools intersect (v2.25.0) to exclude reads which aligned outside of the +15th to −10th codon of the CDS, counted reads using RSEM (rsem-calculate-expression --alignments --strandedness forward --seed-length 20 --sampling-for-bam). Gene counts were then normalized and analyzed by DESeq2^27,44^. Three replicates were produced per condition. DESeq2 normalized (median-of-ratios) gene-level counts for each replicated condition were averaged. mRNA sequencing libraries were processed by an identical pipeline and normalized and analyzed by DESeq2. To compare ribosome profiling and irCLIP datasets, the irCLIP reads were re-processed by the same pipeline for ribosome profiling libraries, but only the irCLIP read counts to the 3’-UTR region of genes were analyzed by DESeq2.

For initiation, termination, and solitary codon occupancy profiles, only genes with ≥ 50 nt of annotated 5ʹ-UTR, ≥ 450 nt of annotated CDS, ≥ 50 nt of annotated 3ʹ-UTR, and ≥ 1 reads per codon were considered. Alignments were counted based on read length and either 5ʹ or 3ʹ position relative to the first base of a codon of interest (e.g., start codon), and rescaled by average number of reads per codon for a given gene. Rescaled counts for each read length and relative position were summed and divided by the number of genes under consideration. For visualization, read lengths were binned (e.g., read lengths of 58, 59, and 60 nt were binned to 58 – 60 nt) and summed by rescaled counts. A-site codon offsets for P1 nuclease footprints were assigned based on the frame of the 5ʹ end of the read, as described in^24^.

## Supplementary Material

Supplement 1

Supplement 2

Supplement 3

Supplement 4

## Data Availability

Data underlying this article are publicly available. irCLIP-seq and RNA-seq data are deposited under GEO accession GSE246727. Sequencing data from the APA-seq experiment are available as GEO accession GSE246786. Ribosome profiling data are accessible as SRA project ID PRJNAXXXXXXX. Scripts for data processing are available upon request.

## References

[1] Saba J. A., Liakath-Ali K., Green R. & Watt F. M. Translational control of stem cell function. Nat. Rev. Mol. Cell Biol. 22, 671–690 (2021). 34272502 10.1038/s41580-021-00386-2

[2] Sampath P. A Hierarchical Network Controls Protein Translation during Murine Embryonic Stem Cell Self-Renewal and Differentiation. Cell Stem Cell 2, 448–460 (2008). 18462695 10.1016/j.stem.2008.03.013

[3] Signer R. A. J., Magee J. A., Salic A. & Morrison S. J. Haematopoietic stem cells require a highly regulated protein synthesis rate. Nature 509, 49–54 (2014). 24670665 10.1038/nature13035 PMC4015626

[4] Baser A. Onset of differentiation is post-transcriptionally controlled in adult neural stem cells. Nature 566, 100–104 (2019). 30700908 10.1038/s41586-019-0888-x

[5] Blanco S. Stem cell function and stress response are controlled by protein synthesis. Nature 534, 335–340 (2016). 27306184 10.1038/nature18282 PMC5040503

[6] Zismanov V. Phosphorylation of eIF2α Is a Translational Control Mechanism Regulating Muscle Stem Cell Quiescence and Self-Renewal. Cell Stem Cell 18, 79–90 (2016). 26549106 10.1016/j.stem.2015.09.020

[7] Jackson R. J., Hellen C. U. T. & Pestova T. V. The mechanism of eukaryotic translation initiation and principles of its regulation. Nat. Rev. Mol. Cell Biol. 11, 113–127 (2010). 20094052 10.1038/nrm2838 PMC4461372

[8] Hafner M. Transcriptome-wide Identification of RNA-Binding Protein and MicroRNA Target Sites by PAR-CLIP. Cell 141, 129–141 (2010). 20371350 10.1016/j.cell.2010.03.009 PMC2861495

[9] Lee A. S. Y., Kranzusch P. J. & Cate J. H. D. eIF3 targets cell-proliferation messenger RNAs for translational activation or repression. Nature 522, 111–114 (2015). 25849773 10.1038/nature14267 PMC4603833

[10] Lee A. S. Y., Kranzusch P. J., Doudna J. A. & Cate J. H. D. eIF3d is an mRNA cap-binding protein that is required for specialized translation initiation. Nature 536, 96–99 (2016). 27462815 10.1038/nature18954 PMC5003174

[11] De Silva D. Robust T cell activation requires an eIF3-driven burst in T cell receptor translation. eLife 10, e74272 (2021). 34970966 10.7554/eLife.74272 PMC8758144

[12] Chambers S. M. Highly efficient neural conversion of human ES and iPS cells by dual inhibition of SMAD signaling. Nat. Biotechnol. 27, 275–280 (2009). 19252484 10.1038/nbt.1529 PMC2756723

[13] Kaczynski T., Hussain A. & Farkas M. Quick-irCLIP: Interrogating protein-RNA interactions using a rapid and simple cross-linking and immunoprecipitation technique. MethodsX 6, 1292–1304 (2019). 31205862 10.1016/j.mex.2019.05.014 PMC6558091

[14] Szklarczyk D. STRING v11: protein–protein association networks with increased coverage, supporting functional discovery in genome-wide experimental datasets. Nucleic Acids Res. 47, D607–D613 (2019). 30476243 10.1093/nar/gky1131 PMC6323986

[15] Heinz S. Simple Combinations of Lineage-Determining Transcription Factors Prime cis-Regulatory Elements Required for Macrophage and B Cell Identities. Mol. Cell 38, 576–589 (2010). 20513432 10.1016/j.molcel.2010.05.004 PMC2898526

[16] Tushev G. Alternative 3′ UTRs Modify the Localization, Regulatory Potential, Stability, and Plasticity of mRNAs in Neuronal Compartments. Neuron 98, 495–511.e6 (2018). 29656876 10.1016/j.neuron.2018.03.030

[17] Taliaferro J. M. Distal Alternative Last Exons Localize mRNAs to Neural Projections. Mol. Cell 61, 821–833 (2016). 26907613 10.1016/j.molcel.2016.01.020 PMC4798900

[18] Miura P., Shenker S., Andreu-Agullo C., Westholm J. O. & Lai E. C. Widespread and extensive lengthening of 3’ UTRs in the mammalian brain. Genome Res. 23, 812–825 (2013). 23520388 10.1101/gr.146886.112 PMC3638137

[19] Ludwin S. K., Kosek J. C. & Eng L. F. The topographical distribution of S-100 and GFA proteins in the adult rat brain: An immunohistochemical study using horseradish peroxidase-labelled antibodies. J. Comp. Neurol. 165, 197–207 (1976). 1107363 10.1002/cne.901650206

[20] Brockes J. P., Fields K. L. & Raff M. C. Studies on cultured rat Schwann cells. I. Establishment of purified populations from cultures of peripheral nerve. Brain Res. 165, 105–118 (1979). 371755 10.1016/0006-8993(79)90048-9

[21] Ferri G.-L. Evidence for the presence of S-100 protein in the glial component of the human enteric nervous system. Nature 297, 409–410 (1982). 7043279 10.1038/297409a0

[22] Havrda M. C. Id2 Is Required for Specification of Dopaminergic Neurons during Adult Olfactory Neurogenesis. J. Neurosci. 28, 14074–14087 (2008). 19109490 10.1523/JNEUROSCI.3188-08.2008 PMC2692601

[23] Ingolia N. T., Lareau L. F. & Weissman J. S. Ribosome Profiling of Mouse Embryonic Stem Cells Reveals the Complexity and Dynamics of Mammalian Proteomes. Cell 147, 789–802 (2011). 22056041 10.1016/j.cell.2011.10.002 PMC3225288

[24] Ferguson L., Streamlined and sensitive mono- and diribosome profiling in yeast and human cells. Nat Methods. 1–12 (2023). 37783882 10.1038/s41592-023-02028-1 PMC11276118

[25] Upton H. E. Low-bias ncRNA libraries using ordered two-template relay: Serial template jumping by a modified retroelement reverse transcriptase. Proc. Natl. Acad. Sci. 118, e2107900118 (2021). 34649994 10.1073/pnas.2107900118 PMC8594491

[26] Meydan S. & Guydosh N. R. Disome and Trisome Profiling Reveal Genome-wide Targets of Ribosome Quality Control. Mol. Cell 79, 588–602.e6 (2020). 32615089 10.1016/j.molcel.2020.06.010 PMC7484464

[27] Love M. I., Huber W. & Anders S. Moderated estimation of fold change and dispersion for RNA-seq data with DESeq2. Genome Biol. 15, 550 (2014). 25516281 10.1186/s13059-014-0550-8 PMC4302049

[28] Martineau Y. Poly(A)-Binding Protein-Interacting Protein 1 Binds to Eukaryotic Translation Initiation Factor 3 To Stimulate Translation. Mol. Cell. Biol. 28, 6658–6667 (2008). 18725400 10.1128/MCB.00738-08 PMC2573229

[29] Wells S. E., Hillner P. E., Vale R. D. & Sachs A. B. Circularization of mRNA by Eukaryotic Translation Initiation Factors. Mol. Cell 2, 135–140 (1998). 9702200 10.1016/s1097-2765(00)80122-7

[30] Imataka H. A newly identified N-terminal amino acid sequence of human eIF4G binds poly(A)-binding protein and functions in poly(A)-dependent translation. EMBO J. 17, 7480–7489 (1998). 9857202 10.1093/emboj/17.24.7480 PMC1171091

[31] Borman A. M. Biochemical characterisation of cap-poly(A) synergy in rabbit reticulocyte lysates: the eIF4G-PABP interaction increases the functional affinity of eIF4E for the capped mRNA 5’-end. Nucleic Acids Res. 28, 4068–4075 (2000). 11058101 10.1093/nar/28.21.4068 PMC113128

[32] Kahvejian A., Svitkin Y. V., Sukarieh R., M’Boutchou M.-N. & Sonenberg N. Mammalian poly(A)-binding protein is a eukaryotic translation initiation factor, which acts via multiple mechanisms. Genes Dev. 19, 104–113 (2005). 15630022 10.1101/gad.1262905 PMC540229

[33] Tarun S. Z. & Sachs A. B. Association of the yeast poly(A) tail binding protein with translation initiation factor eIF-4G. EMBO J. 15, 7168–7177 (1996). 9003792 PMC452544

[34] Choe J. mRNA circularization by METTL3–eIF3h enhances translation and promotes oncogenesis. Nature 561, 556–560 (2018). 30232453 10.1038/s41586-018-0538-8 PMC6234840

[35] abc de dummyref

[36] Aleksashin N., Chang S. T.-L. & Cate J. A highly efficient human cell-free translation system. *RNA* rna.079825.123 (2023) doi:10.1261/rna.079825.123. PMC10653386 37793791

[37] Martin M. Cutadapt removes adapter sequences from high-throughput sequencing reads. EMBnet.journal 17, 10–12 (2011).

[38] Ramírez F. deepTools2: a next generation web server for deep-sequencing data analysis. Nucleic Acids Res. 44, W160–W165 (2016). 27079975 10.1093/nar/gkw257 PMC4987876

[39] Twelve years of SAMtools and BCFtools | GigaScience | Oxford Academic. https://academic.oup.com/gigascience/article/10/2/giab008/6137722. 10.1093/gigascience/giab008 PMC7931819 33590861

[40] Kim D., Paggi J. M., Park C., Bennett C. & Salzberg S. L. Graph-based genome alignment and genotyping with HISAT2 and HISAT-genotype. Nat. Biotechnol. 37, 907–915 (2019). 31375807 10.1038/s41587-019-0201-4 PMC7605509

[41] Li H. The Sequence Alignment/Map format and SAMtools. Bioinforma. Oxf. Engl. 25, 2078–2079 (2009). 10.1093/bioinformatics/btp352 PMC2723002 19505943

[42] McGlincy N. J. & Ingolia N. T. Transcriptome-wide measurement of translation by ribosome profiling. Methods San Diego Calif 126, 112–129 (2017). 28579404 10.1016/j.ymeth.2017.05.028 PMC5582988

[43] Holmes A. D., Howard J. M., Chan P. P. & Lowe T. M. tRNA Analysis of eXpression (tRAX): A tool for integrating analysis of tRNAs, tRNA-derived small RNAs, and tRNA modifications. 2022.07.02.498565 Preprint at 10.1101/2022.07.02.498565 (2022).

[44] Li B., Dewey C. N. RSEM: accurate transcript quantification from RNA-Seq data with or without a reference genome. BMC Bioinformatics 12, (2011). 10.1186/1471-2105-12-323 PMC3163565 21816040

