# Supplementary material for: eIF3 engages with 3’-UTR termini of highly translated mRNAs
Source: bioRxiv. 2024 Nov 24:2023.11.11.566681. Originally published 2023 Nov 11. Preprint. [Version 4] doi: 10.1101/2023.11.11.566681 (PMC10659435; doi:10.1101/2023.11.11.566681)
Supplement: Supplement 7 [file media-7.pdf]

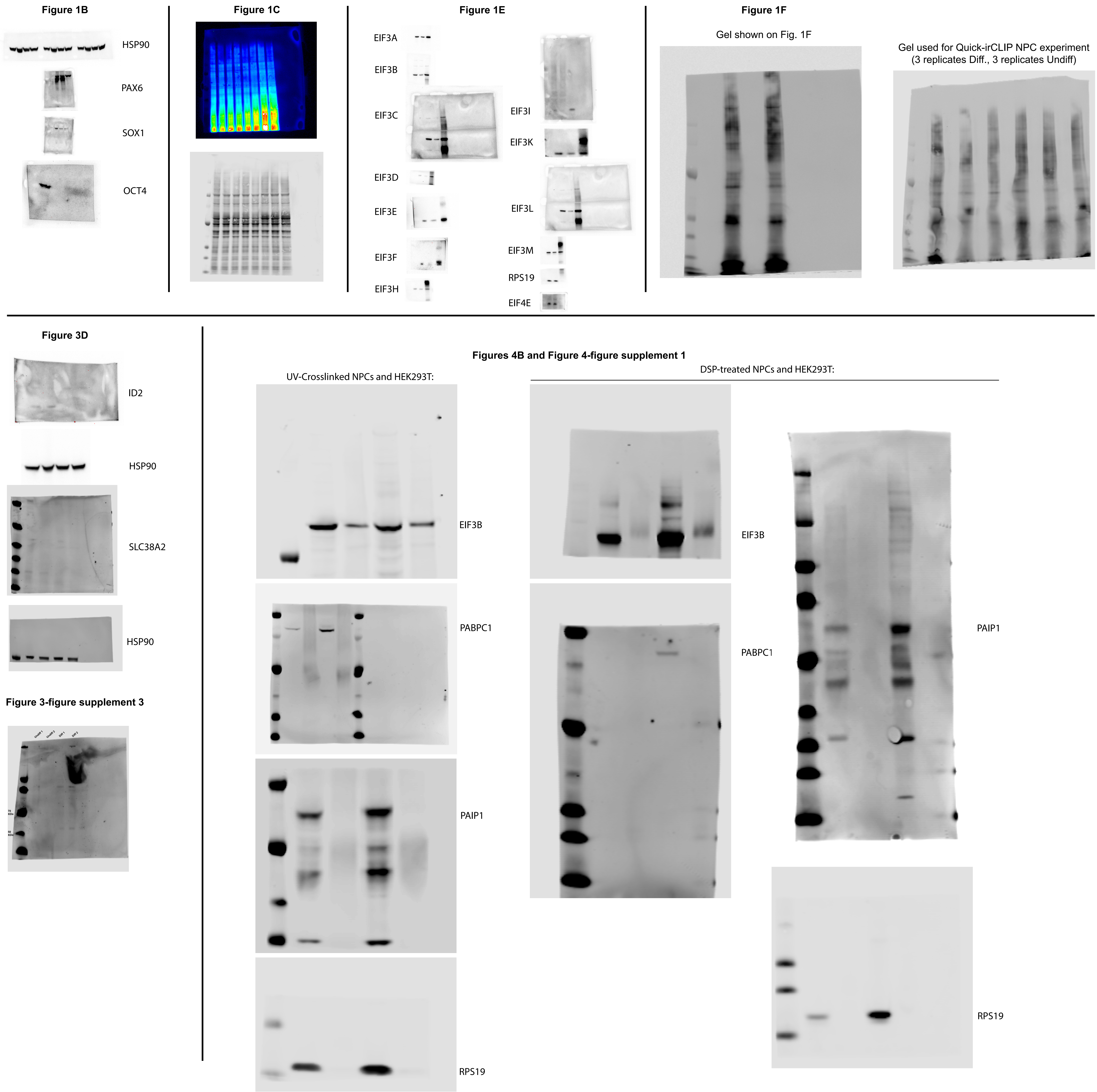

Figure 1B
HSP90
PAX6
SOX1
OCT4
Figure 1C
Figure 1E
EIF3A
EIF3B
EIF3C
EIF3D
EIF3E
EIF3F
EIF3H
EIF3I
EIF3K
EIF3L
EIF3M
RPS19
EIF4E
Figure 1F
Gel shown on Fig. 1F
Gel used for Quick-irCLIP NPC experiment
(3 replicates Diff., 3 replicates Undiff)
Figure 3D
ID2
HSP90
SLC38A2
HSP90
Figure 3-figure supplement 3
Figures 4B and Figure 4-figure supplement 1
UV-Crosslinked NPCs and HEK293T:
EIF3B
PABPC1
PAIP1
RPS19
DSP-treated NPCs and HEK293T:
EIF3B
PABPC1
PAIP1
RPS19
