# Supplementary material for: eIF3 engages with 3’-UTR termini of highly translated mRNAs in neural progenitor cells
Source: bioRxiv. 2023 Nov 11:2023.11.11.566681. Preprint. [Version 1] doi: 10.1101/2023.11.11.566681 (PMC10659435; doi:10.1101/2023.11.11.566681)
Supplement: Supplementary file 1 [file NIHPP2023.11.11.566681v1-supplement-4.pdf]

bioRxiv preprint doi: https://doi.org/10.1101/2023.11.11.566681; this version posted November 11, 2023. The copyright holder for this preprint (which was not certified by peer review) is the author/funder, who has granted bioRxiv a license to display the preprint in perpetuity. It is made available under aCC-BY 4.0 International license.

SUPPLEMENTARY INFORMATION

eIF3 engages with 3'-UTR termini of highly translated mRNAs in neural progenitor cells

Santi Mestre-Fos[1,2], Lucas Ferguson[2,3], Marena Trinidad[1,2], Nicholas T. Ingolia[2,4], Jamie H. D. Cate[1,2,5]

[1]Innovative Genomics Institute, University of California, Berkeley, CA, USA.

[2]Department of Molecular and Cell Biology, University of California, Berkeley, CA, USA.

[3]Center for Computational Biology, University of California, Berkeley, CA, USA

[4]California Institute for Quantitative Biosciences, University of California, Berkeley, CA, USA

[5]Department of Chemistry, University of California, Berkeley, CA, USA.

bioRxiv preprint doi: https://doi.org/10.1101/2023.11.11.566681; this version posted November 11, 2023. The copyright holder for this preprint (which was not certified by peer review) is the author/funder, who has granted bioRxiv a license to display the preprint in perpetuity. It is made available under aCC-BY 4.0 International license.

Supplementary Tables

Table S1. Subunits of eIF3 and proteins pulled down by anti-EIF3B IP. The IP was performed using lysates from undifferentiated NPCs. Given are protein names, sequence coverage, and number of peptides detected.

Table S2. Annotation of clusters from Quick-irCLIP of eIF3 to RNAs in undifferentiated NPCs. Three different filters for conflicts in gene annotation are presented. Filter 0 prioritizes "mRNA" as an annotation. Filter 1 prioritizes the 3'-UTR in mRNA annotations. Filter 2 prioritizes the 5'-UTR in mRNA annotations. Figure 1 and Figure 2C present results using Filter 1.

Table S3. Annotation of clusters from Quick-irCLIP of eIF3 to RNAs in differentiated NPCs. Three different filters for conflicts in gene annotation are presented. Filter 0 prioritizes "mRNA" as an annotation. Filter 1 prioritizes the 3'-UTR in mRNA annotations. Filter 2 prioritizes the 5'-UTR in mRNA annotations. Figure 1 and Figure 2C present results using Filter 1.

bioRxiv preprint doi: https://doi.org/10.1101/2023.11.11.566681; this version posted November 11, 2023. The copyright holder for this preprint (which was not certified by peer review) is the author/funder, who has granted bioRxiv a license to display the preprint in perpetuity. It is made available under aCC-BY 4.0 International license.

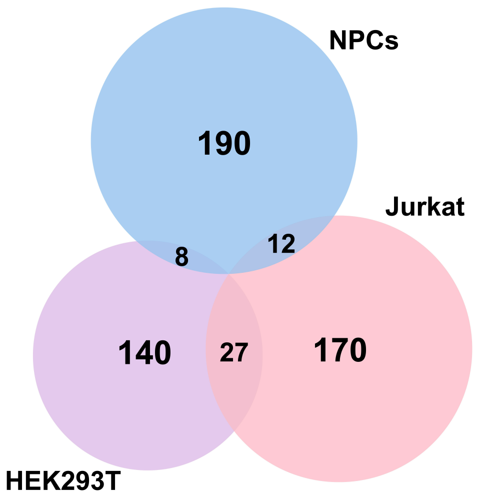

Figure S1. Venn Diagram of genes identified by Quick-irCLIP (differentiated NPCs) and PAR-CLIP (HEK293T and activated Jurkat T cells). The top ~ 200 mRNA transcripts from the three libraries ranked by total reads mapped to a given gene are compared (See Materials and Methods).

bioRxiv preprint doi: https://doi.org/10.1101/2023.11.11.566681; this version posted November 11, 2023. The copyright holder for this preprint (which was not certified by peer review) is the author/funder, who has granted bioRxiv a license to display the preprint in perpetuity. It is made available under aCC-BY 4.0 International license.

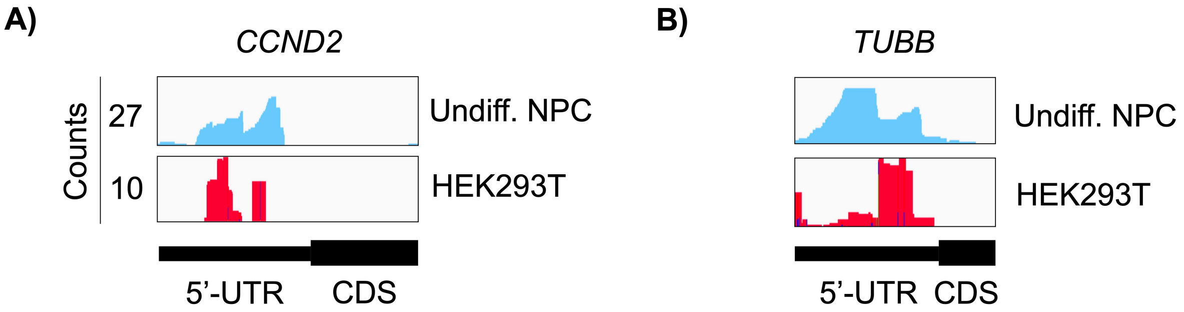

Figure S2. Crosslinking of eIF3 in undifferentiated NPCs and in HEK293T cells. Crosslinks in undifferentiated NPCs are in blue, and in HEK294T cells in red. Shown are the 5'-UTR regions of **A)** *CCND2* and **B)** *TUBB* mRNAs.

bioRxiv preprint doi: https://doi.org/10.1101/2023.11.11.566681; this version posted November 11, 2023. The copyright holder for this preprint (which was not certified by peer review) is the author/funder, who has granted bioRxiv a license to display the preprint in perpetuity. It is made available under aCC-BY 4.0 International license.

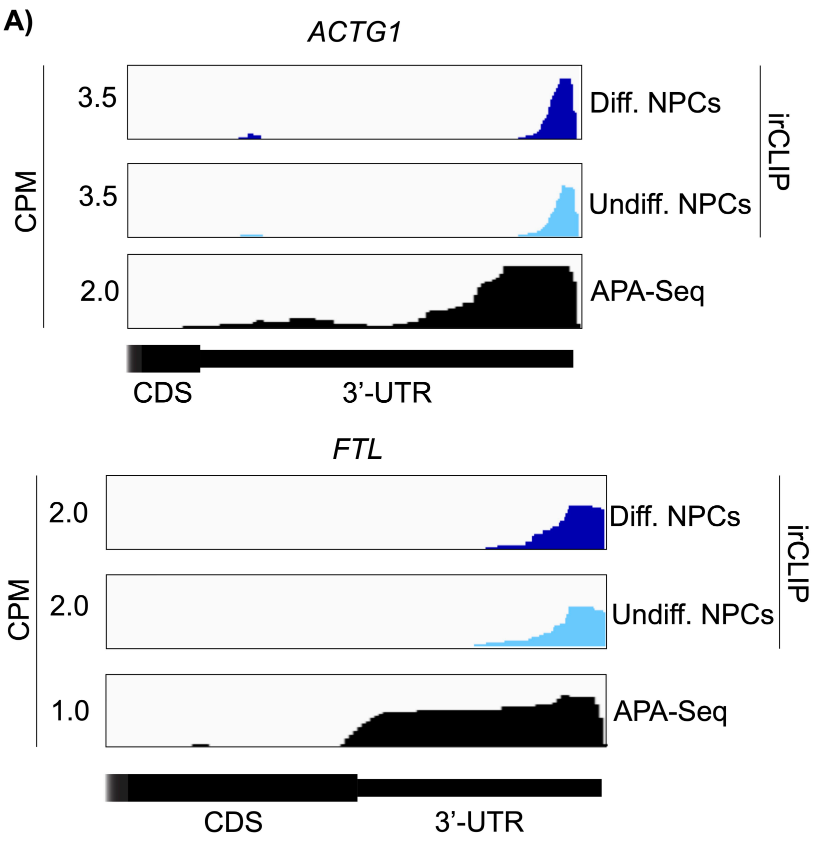

Figure S3. Crosslinking of eIF3 in differentiated and undifferentiated NPCs across the 3'-UTR regions of *ACTG1* and *FTL* mRNAs. APA-Seq data is also shown for both transcripts.

bioRxiv preprint doi: https://doi.org/10.1101/2023.11.11.566681; this version posted November 11, 2023. The copyright holder for this preprint (which was not certified by peer review) is the author/funder, who has granted bioRxiv a license to display the preprint in perpetuity. It is made available under aCC-BY 4.0 International license.

Undifferentiated NPCs:

* - possible false positive

Rank	Motif	P-value	log P-pvalue	% of Targets	% of Background
1		1e-929	-2.141e+03	55.07%	16.79%
2		1e-41	-9.637e+01	10.17%	5.56%
3		1e-36	-8.349e+01	11.92%	7.19%
4		1e-34	-8.003e+01	28.97%	21.93%
5		1e-34	-7.862e+01	2.86%	0.92%
6		1e-23	-5.352e+01	1.55%	0.42%
7		1e-18	-4.152e+01	0.99%	0.23%
8		1e-17	-4.025e+01	3.62%	1.87%
9		1e-17	-3.977e+01	4.94%	2.85%
10		1e-16	-3.911e+01	1.31%	0.40%
11		1e-16	-3.827e+01	0.99%	0.25%
12		1e-16	-3.779e+01	10.86%	7.73%
13		1e-15	-3.614e+01	0.49%	0.06%
14		1e-15	-3.503e+01	6.25%	4.00%
15		1e-14	-3.340e+01	2.45%	1.17%
16		1e-13	-3.023e+01	0.16%	0.00%
17		1e-12	-2.863e+01	0.37%	0.04%
18		1e-12	-2.764e+01	3.11%	1.75%
19 *		1e-11	-2.760e+01	1.32%	0.52%
20 *		1e-10	-2.500e+01	0.58%	0.14%
21 *		1e-10	-2.495e+01	1.13%	0.43%
22 *		1e-10	-2.331e+01	0.32%	0.04%
23 *		1e-9	-2.211e+01	0.12%	0.00%
24 *		1e-9	-2.210e+01	0.69%	0.21%
25 *		1e-9	-2.159e+01	0.23%	0.02%
26 *		1e-9	-2.128e+01	2.91%	1.75%
27 *		1e-8	-1.964e+01	1.01%	0.42%
28 *		1e-8	-1.906e+01	0.26%	0.04%

Differentiated NPCs:

* - possible false positive

Rank	Motif	P-value	log P-pvalue	% of Targets	% of Background	STD(Bg STD)
1		1e-792	-1.825e+03	58.54%	17.12%	15.5bp (30.0bp)
2		1e-33	-7.728e+01	30.66%	22.53%	23.4bp (28.4bp)
3		1e-33	-7.659e+01	9.67%	5.08%	18.4bp (31.3bp)
4		1e-29	-6.890e+01	6.22%	2.84%	22.3bp (25.0bp)
5		1e-25	-5.854e+01	2.71%	0.84%	25.3bp (21.2bp)
6		1e-24	-5.709e+01	3.06%	1.05%	23.6bp (24.3bp)
7		1e-18	-4.284e+01	4.61%	2.27%	23.9bp (24.7bp)
8		1e-18	-4.157e+01	7.65%	4.56%	24.6bp (25.5bp)
9		1e-17	-3.986e+01	0.81%	0.11%	19.2bp (16.4bp)
10		1e-16	-3.727e+01	0.90%	0.16%	21.0bp (21.4bp)
11		1e-15	-3.489e+01	0.26%	0.01%	13.9bp (18.5bp)
12		1e-13	-3.194e+01	1.76%	0.62%	22.8bp (24.3bp)
13		1e-12	-2.949e+01	0.95%	0.23%	22.6bp (19.9bp)
14		1e-12	-2.875e+01	0.19%	0.00%	26.1bp (9.3bp)
15		1e-12	-2.875e+01	0.19%	0.00%	19.5bp (0.0bp)
16		1e-12	-2.842e+01	1.42%	0.48%	21.6bp (21.8bp)
17 *		1e-11	-2.690e+01	0.33%	0.02%	21.2bp (17.9bp)
18 *		1e-10	-2.507e+01	2.23%	1.03%	23.5bp (23.6bp)
19 *		1e-10	-2.426e+01	1.76%	0.74%	21.4bp (22.7bp)
20 *		1e-10	-2.419e+01	0.24%	0.01%	13.8bp (10.7bp)
21 *		1e-9	-2.158e+01	0.95%	0.30%	23.1bp (26.8bp)
22 *		1e-8	-1.965e+01	0.17%	0.01%	9.6bp (0.0bp)
23 *		1e-7	-1.755e+01	0.21%	0.02%	16.2bp (21.9bp)
24 *		1e-7	-1.614e+01	0.14%	0.01%	14.5bp (6.3bp)
25 *		1e-6	-1.546e+01	0.85%	0.32%	17.1bp (24.5bp)
26 *		1e-5	-1.278e+01	0.12%	0.01%	22.0bp (11.9bp)
27 *		1e-5	-1.278e+01	0.12%	0.01%	18.7bp (1.8bp)
28 *		1e-5	-1.255e+01	0.74%	0.29%	21.1bp (17.2bp)
29 *		1e-5	-1.232e+01	0.17%	0.02%	21.7bp (13.6bp)

Figure S4. Motif enrichment of eIF3 3’-UTR crosslinks for undifferentiated and differentiated NPCs. Motifs were identified with HOMER (v4.11).

bioRxiv preprint doi: https://doi.org/10.1101/2023.11.11.566681; this version posted November 11, 2023. The copyright holder for this preprint (which was not certified by peer review) is the author/funder, who has granted bioRxiv a license to display the preprint in perpetuity. It is made available under aCC-BY 4.0 International license.

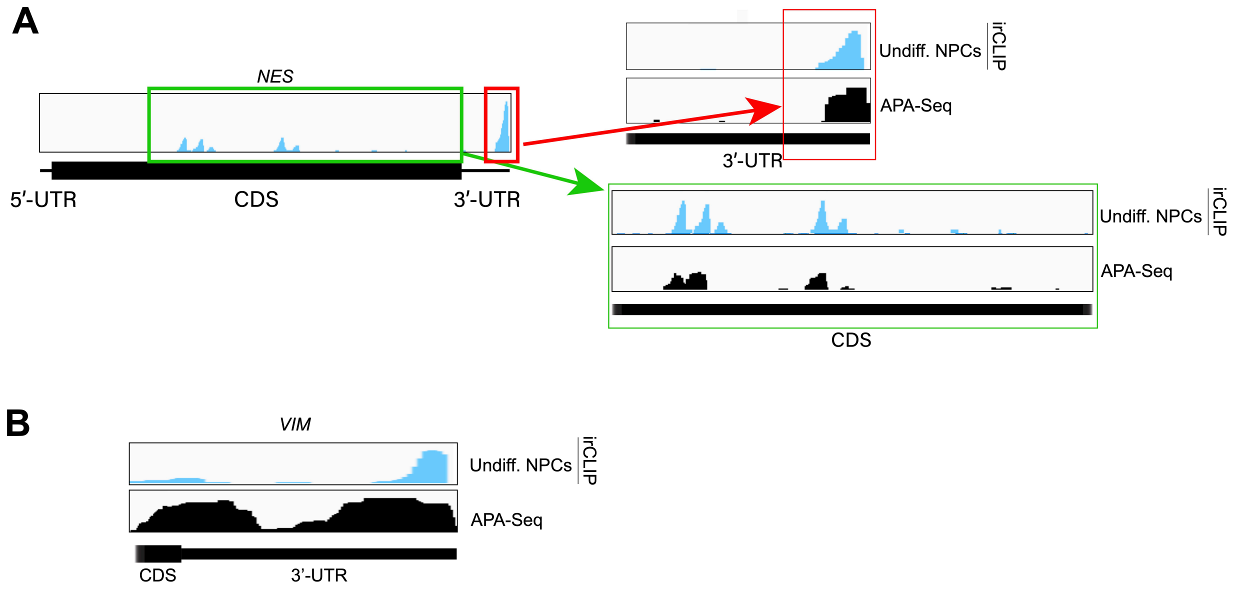

Figure S5. Examples of eIF3 crosslinking to 3'-UTR regions of mRNAs. A) Crosslinking of eIF3 in undifferentiated NPCs as indicated across the 3'-UTR region of *NES* mRNA. APA-Seq data is also shown. **B)** Crosslinking of eIF3 in undifferentiated NPCs as indicated across the 3'-UTR region of *VIM* mRNA. APA-Seq data is also shown.

bioRxiv preprint doi: https://doi.org/10.1101/2023.11.11.566681; this version posted November 11, 2023. The copyright holder for this preprint (which was not certified by peer review) is the author/funder, who has granted bioRxiv a license to display the preprint in perpetuity. It is made available under aCC-BY 4.0 International license.

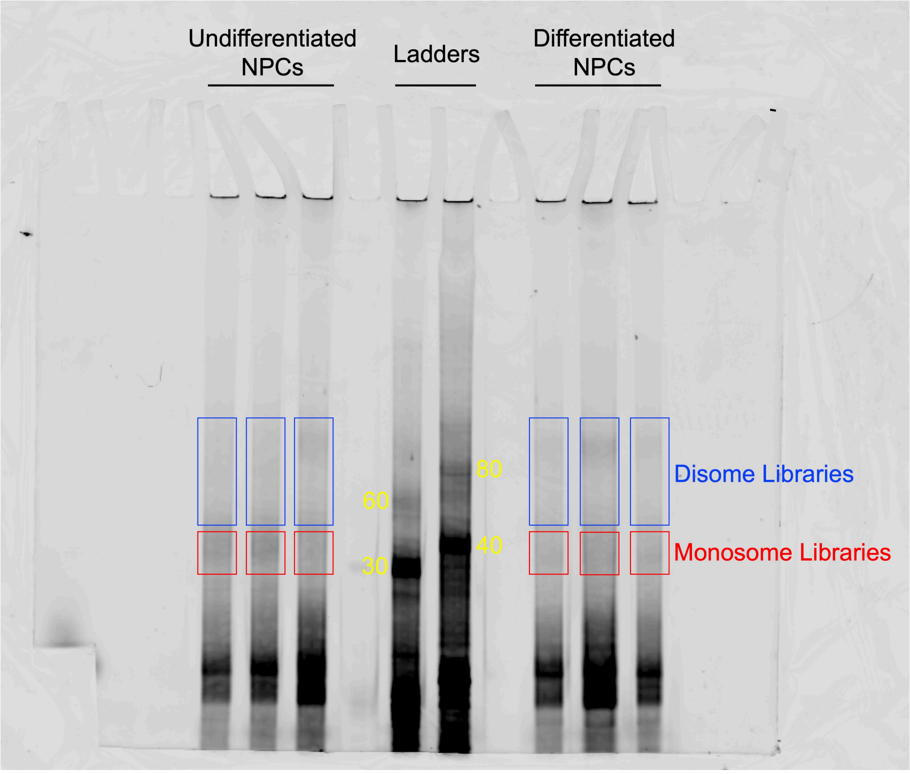

Figure S6. TBE 8% Urea-PAGE of the OTTR reaction products of cDNA libraries constructed from undifferentiated and differentiated NPCs. OTTR was also performed with control samples that yield marker products of 30, 40, 60, 80 nts in size (marked in yellow) so they could be used as references for gel excision for monosome and disome libraries. Gel excisions performed are shown in red (monosome) and blue (disome) rectangles.

bioRxiv preprint doi: https://doi.org/10.1101/2023.11.11.566681; this version posted November 11, 2023. The copyright holder for this preprint (which was not certified by peer review) is the author/funder, who has granted bioRxiv a license to display the preprint in perpetuity. It is made available under aCC-BY 4.0 International license.

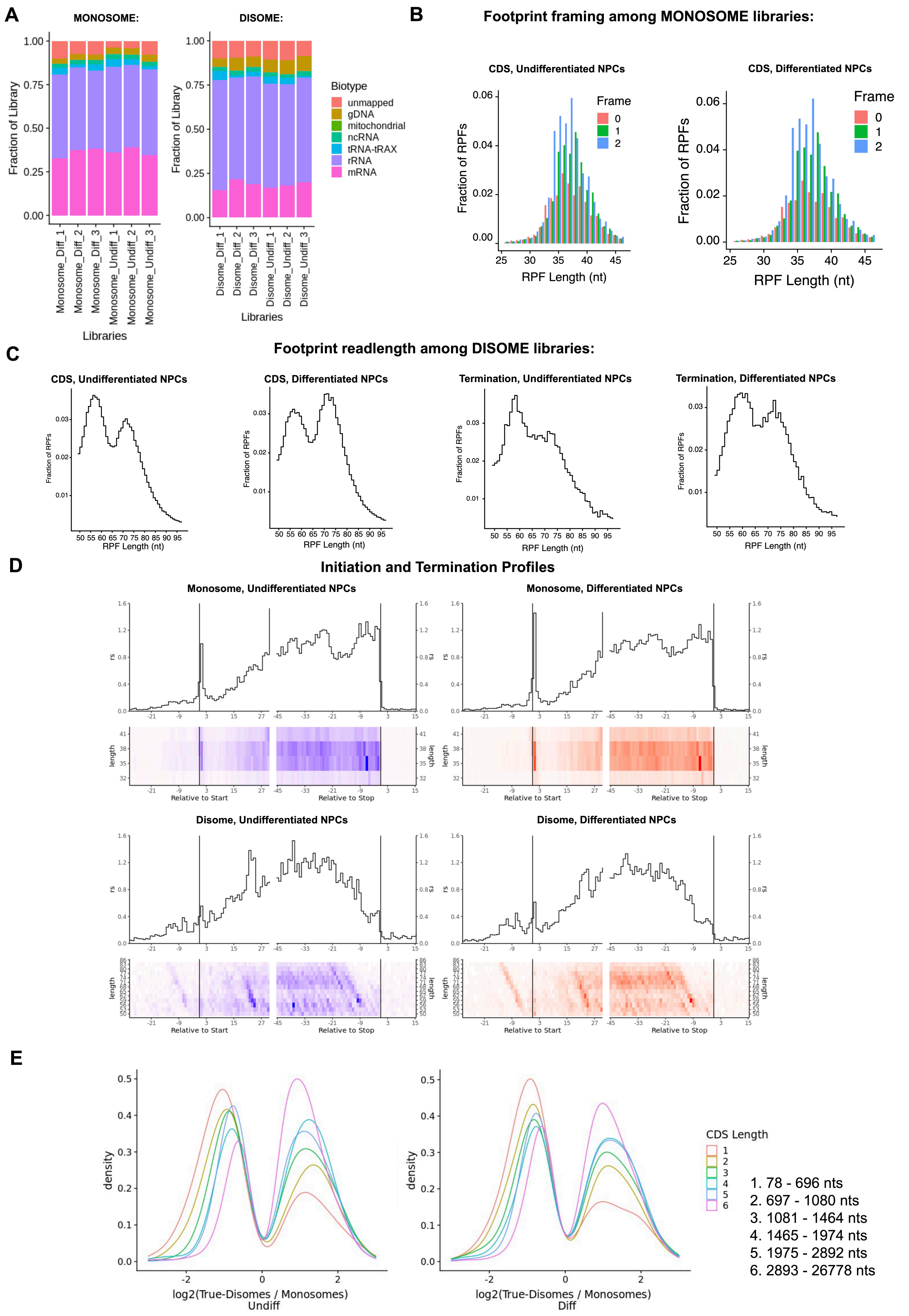

bioRxiv preprint doi: https://doi.org/10.1101/2023.11.11.566681; this version posted November 11, 2023. The copyright holder for this preprint (which was not certified by peer review) is the author/funder, who has granted bioRxiv a license to display the preprint in perpetuity. It is made available under aCC-BY 4.0 International license.

Figure S7. Ribosome profiling of undifferentiated and differentiated NPCs. A) Fraction of sequencing reads mapped to each transcript type from monosome and disome profiling from P1 nuclease digested NPCs (undifferentiated and differentiated) and OTTR library cDNA synthesis. **B)** Framing among monosome libraries in undifferentiated and differentiated NPCs. **C)** Readlength of footprints aligned to CDS or terminating codons among disome libraries for undifferentiated and differentiated NPCs. **D)** A-site corrected initiation and termination profiles for genes with sufficient coverage (≥1 read per codon) for monosome and disome libraries. **E)** DESeq2 log2 fold change in CDS occupancy for disome libraries with respect to monosome libraries, stratified by CDS length in nucleotides.

bioRxiv preprint doi: https://doi.org/10.1101/2023.11.11.566681; this version posted November 11, 2023. The copyright holder for this preprint (which was not certified by peer review) is the author/funder, who has granted bioRxiv a license to display the preprint in perpetuity. It is made available under aCC-BY 4.0 International license.

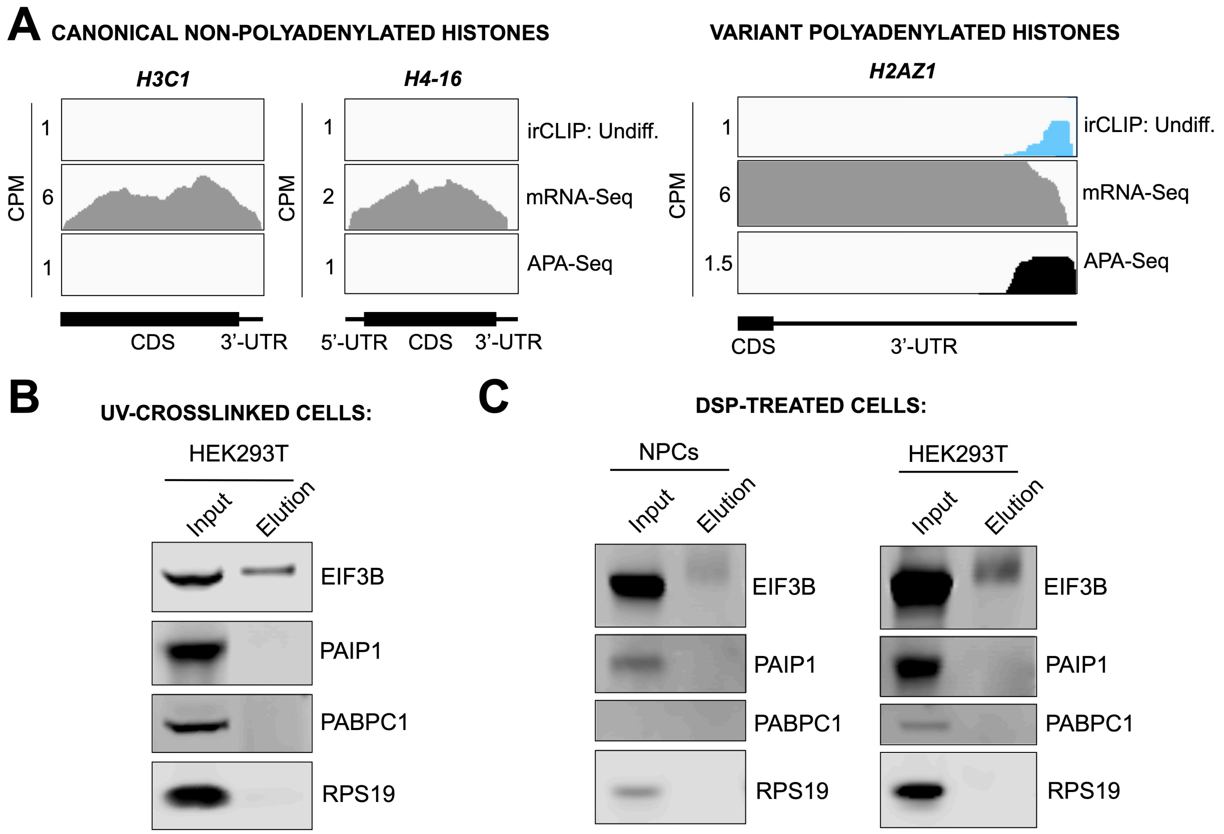

Figure S8. Crosslinking of eIF3 to histone mRNAs in NPCs. A) Crosslinking of eIF3 in undifferentiated NPCs as indicated across the canonical non-polyadenylated histone *H3C1* and *H4-16* mRNAs and across the variant polyadenylated histone *H2AZ1* mRNA. Western blots for EIF3B immunoprecipitation samples from **B)** UV-crosslinked HEK293T and **C)** DSP-treated undifferentiated NPCs and HEK293T.

bioRxiv preprint doi: https://doi.org/10.1101/2023.11.11.566681; this version posted November 11, 2023. The copyright holder for this preprint (which was not certified by peer review) is the author/funder, who has granted bioRxiv a license to display the preprint in perpetuity. It is made available under aCC-BY 4.0 International license.

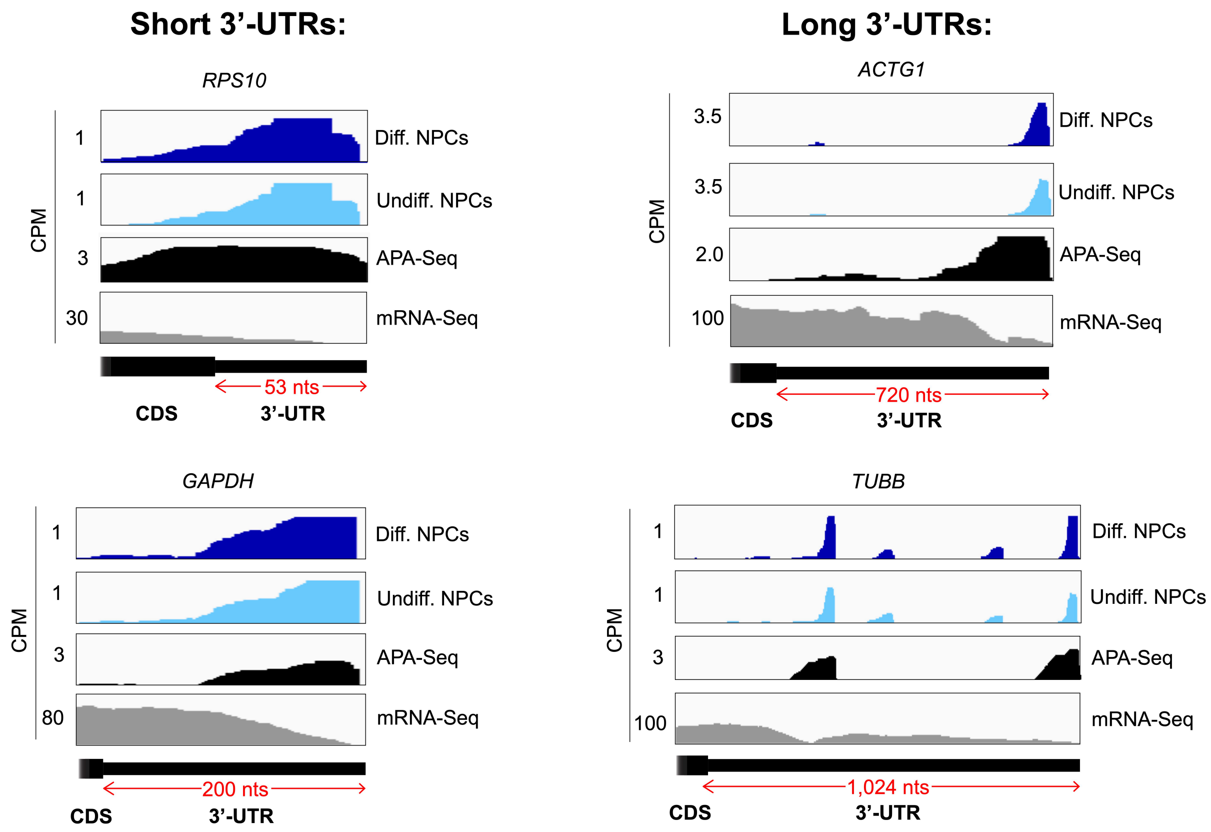

Figure S9. Crosslinking of eIF3 to mRNA 3'-UTRs of various lengths in NPCs. Crosslinking patterns of eIF3 in differentiated and undifferentiated NPCs across *RPS10*, *GAPDH*, *ACTG1*, and *TUBB* mRNAs are shown. The length of each 3'-UTR is indicated. APA-Seq and mRNA-Seq data are also shown.
